# An integrative approach for studying immunological variation in an aging population – The *Milieu Intérieur* follow-up study

**DOI:** 10.1038/s41467-026-72910-x

**Published:** 2026-05-22

**Authors:** Anthony Jaquaniello, Florian Dubois, Farah Rahal, Tom Dott, Slobodan Culina, Chloe Albert-Vega, Mélanie Briard, Vincent Rouilly, Etienne Villain, Violaine Saint-André, Divya Unni, Sandrine Fernandes Pellerin, Emma Bloch, Michael White, Milena Hasan, Lluis Quintana-Murci, Etienne Patin, Darragh Duffy, Milieu Intérieur Consortium, Milena Hasan, Lluis Quintana-Murci, Etienne Patin, Darragh Duffy, Laurent Abel, Andres Alcover, Hugues Aschard, Philippe Bousso, Nollaig Bourke, Petter Brodin, Pierre Bruhns, Nadine Cerf-Bensussan, Ana Cumano, Christophe D’Enfert, Caroline Demangel, Ludovic Deriano, Marie-Agnès Dillies, James Di Santo, Gérard Eberl, Jost Enninga, Jacques Fellay, Ivo Gomperts-Boneca, Gunilla Karlsson Hedestam, Serge Hercberg, Molly A. Ingersoll, Olivier Lantz, Rose Anne Kenny, Mickaël Ménager, Frédérique Michel, Hugo Mouquet, Cliona O’Farrelly, Antonio Rausell, Frédéric Rieux-Laucat, Lars Rogge, Magnus Fontes, Anavaj Sakuntabhai, Olivier Schwartz, Benno Schwikowski, Spencer Shorte, Frédéric Tangy, Antoine Toubert, Mathilde Touvier, Marie-Noëlle Ungeheuer, Christophe Zimmer, Matthew L. Albert

**Affiliations:** 1 Human Evolutionary Genetics Unit, Institut Pasteur, Université Paris Cité, CNRS UMR2000, Paris, France; 2 Data Management Platform, Institut Pasteur, Paris, France; 3 Translational Immunology Unit, Department of Immunology, Institut Pasteur, Université Paris Cité, Paris, France; 4 Single Cell Biomarkers UTechS, Institut Pasteur, Université Paris Cité, Paris, France; 5 DATACTIX, Paris, France; 6 Bioinformatics and Biostatistics HUB, Institut Pasteur, Université Paris Cité, Paris, France; 7 Clinical Research Coordination Office, Institut Pasteur, Paris, France; 8 Infectious Disease Epidemiology and Analytics G5 Unit, Department of Global Health, Institut Pasteur, Université de Paris, Paris, France; 9 Chair Human Genomics and Evolution, Collège de France, Paris, France; 10 Laboratory of Human Genetics of Infectious Diseases, Necker Branch, Inserm U1163, Necker Hospital for Sick Children, Paris, France; 11 St. Giles Laboratory of Human Genetics of Infectious Diseases, Rockefeller Branch, Rockefeller University, New York City, NY USA; 12 Unité Biologie Cellulaire des Lymphocytes, Institut Pasteur, Université Paris Cité, Paris, France; 13 Statistical Genetics Unit, Institut Pasteur, Université Paris Cité, Paris, France; 14 Dynamics of Immune Responses Unit, Institut Pasteur, Université Paris Cité, Paris, France; 15 Trinity Translational Medicine Institute, Trinity College Dublin, Dublin, Ireland; 16 Department of Women’s and Children’s Health, Karolinska Institutet, Stockholm, Sweden; 17 Department of Immunology and Inflammation, Imperial College London, London, UK; 18 Antibodies in Therapy and Pathology Unit, Institut Pasteur, Université Paris Cité, Paris, France; 19 Laboratory of Intestinal Immunity, Imagine Institute, Université Paris Cité, Paris, France; 20 Unit of Lymphocytes and Immunity, Institut Pasteur, Université Paris Cité, Paris, France; 21 Unité Biologie et Pathogénicité Fongiques, Institut Pasteur, Université Paris Cité, Paris, France; 22 Immunobiology and Therapy Unit, Institut Pasteur, Université Paris Cité, Paris, France; 23 Genome Integrity, Immunity and Cancer Unit, Institut Pasteur, Université Paris Cité, Paris, France; 24 Innate Immunity Unit, Institut Pasteur, Université Paris Cité, Inserm, Paris, France; 25 Microenvironment and Immunity Unit, Institut Pasteur, Université Paris Cité, Paris, France; 26 Dynamics of Host-Pathogen Interactions Unit, Institut Pasteur, Université Paris Cité, Paris, France; 27 EPFL, Lausanne, Switzerland; 28 Lausanne University Hospital, Lausanne, Switzerland; 29 Bacterial Cell Wall Genetics Unit, Institut Pasteur, Université Paris Cité, Paris, France; 30 Karolinska Institutet, Stockholm, Sweden; 31 CRESS-EREN, INSERM, Bobigny, France; 32 NACRe Network, Jouy-en-Josas, France; 33 Mucosal Immunology Unit, Institut Pasteur / Institut Cochin, Université Paris Cité, Paris, France; 34 Institut Curie, Paris, France; 35 CIC-BT1428, Institut Curie, Paris, France; 36 Mercer’s Institute for Successful Ageing, Dublin, Ireland; 37 Imagine Institute, Single-Cell Lab, Paris, France; 38 Cytokine Signaling Unit, Institut Pasteur, Université Paris Cité, Paris, France; 39 Humoral Immunology Unit, Institut Pasteur, Université Paris Cité, Paris, France; 40 School of Biochemistry and Immunology, Trinity College Dublin, Dublin, Ireland; 41 School of Medicine, Trinity College Dublin, Dublin, Ireland; 42 Computational Systems Biomedicine Lab, Institut Pasteur, Université Paris Cité, Paris, France; 43 Institut Pasteur–Oncovita Joint Lab, Paris, France; 44 Clinical Genetics Unit, Necker Hospital, Paris, France; 45 Immunoregulation Unit, Institut Pasteur, Université Paris Cité, Paris, France; 46 Institut Roche, Boulogne-Billancourt, France; 47 Ecology and Emergence of Pathogens Unit, Institut Pasteur, Université Paris Cité, Paris, France; 48 University of Tokyo (IMSUT), Tokyo, Japan; 49 Virus and Immunity Unit, Institut Pasteur, Paris, Université, Paris Cité, France; 50 Systems Biology Unit, Institut Pasteur, Université Paris Cité, Paris, France; 51 UTechS Platform, Institut Pasteur, Université Paris Cité, Paris, France; 52 Viral Genetics Unit, Institut Pasteur, Université Paris Cité, Paris, France; 53 Institut de Recherche Saint Louis, Paris, France; 54 ICAReB Biobank, Institut Pasteur, Université Paris Cité, Paris, France; 55 Imaging and Modeling Unit, Institut Pasteur, Université Paris Cité, Paris, France; 56 University of Würzburg, Bioimaging Center, Konstanz, Germany; 57 University of Würzburg, AI & Data Science Center, Kaiserslautern, Germany; 58 Octant Inc., Emeryville, CA USA

**Keywords:** Biomarkers, Translational immunology, Pathogens, Infectious diseases

## Abstract

Human immune responses vary across individuals due to both genetic and environmental factors. We previously established the Milieu Intérieur cohort to define boundaries of healthy immune variation and identify their determinants. To evaluate how immune responses change over time and test whether immune states can predict future disease, we conducted a 10-year follow-up of the cohort. Here we show widespread changes in humoral responses to pathogens over this period, including unexpected seroreversion of cytomegalovirus (CMV) status, despite a general age-related increase in CMV antibodies. We also investigated whether immune profiles at initial recruitment are associated with disease development a decade later. Strikingly, whole blood transcriptional responses to *Staphylococcus aureus* and *Candida albicans* stimulation were predictive of subsequent infectious disease. Collectively, the Milieu Intérieur longitudinal study represents a valuable resource for evaluating immune aging and may help identify predictors of adverse health outcomes.

## Abstract (web summary)

The human immune system and response shows variability between people due to both genetic and environmental factors. Here the authors present a follow up of the Milieu Intérieur study applying an integrative analysis of immunological variation in a defined aging population.

## Introduction

Individuals exhibit substantial variation in terms of disease risk, response to treatments, and clinical outcomes. These differences may arise from naturally-occurring variability in immune responses, which can be due to factors such as age, biological sex, gender, genetic variation, socio-economic status, and environmental exposures^1,2^. Age, in particular, appears to have one of the strongest influences, affecting basic physiological processes through biological aging and changing environmental exposures over time^3–5^. While an increasing number of studies have examined age-related differences in immune responses^6–10^, few have investigated the molecular and cellular mechanisms underlying human immune aging in natural settings^11,12^. The Stanford longitudinal aging study previously reported important, significant inter-individual differences in aging immune responses^13^, but the factors contributing to this phenomenon remain unclear.

To identify the determinants of immune variability, the Milieu Intérieur (MI) population cohort study^14^ was established in 2012–2013, consisting of 1000 well-defined healthy donors equally stratified by age and sex (100 male and 100 female donors in each decade of life between 20 and 69 years old). From these donors, an extensive biocollection of skin, nasal, fecal, and baseline and stimulated blood samples was established, along with comprehensive health and lifestyle information. An ever-growing extensive data warehouse was established, encompassing genomic^15^, epigenomic^16^, transcriptomic^17^, proteomic^18,19^ cellular^15^, serological^20^, and microbiome^21^ datasets for the entire cohort. Using this rich and unique dataset, MI has so far identified and quantified the impact of genetic variants, age, sex, smoking, socioeconomic status, persistent infections, blood group antigens, BMI, and diet on variability in immune cell composition^15^, thymic function^22^, circulating proteins^18^, antibodies and auto-antibodies, and functional immune responses at both baseline levels^20^ and after stimulation with microbes, agonists, and cytokines^17,23–26^.

To address our original questions on understanding immune variability, the MI study was designed as a cross-sectional study with a narrow time frame (with half of the donors resampled within 2–6 weeks), a focus on healthy individuals, and a defined age range (20–69 years of age)^14^. However, such a design precludes the assessment of immune variation with time and beyond 70 years of age, the study of the impact of disease onset on immunity, and the role of age-related immune perturbations in disease development. To address these limitations, we conducted a longitudinal assessment of available donors 10 years after the original study. As the study was not initially designed for longitudinal assessment, anticipating donor participation was challenging. However, following a broad information campaign, we successfully recruited 415 of the original 1000 donors. As we wanted to study disease incidence and outcomes, exclusion criteria were minimal. The same information and biological samples were collected during the new sampling campaign for cross-comparison over the 10-year period. Additionally, given the onset of the COVID-19 pandemic in 2020, we took advantage of this follow-up study to assess the biological underpinnings of immune responses against SARS-CoV-2, by expanding our clinical investigation to coronavirus infections and assessing antibody titers against common coronaviruses at both time-points. Here, we present a description of the 10-year follow-up study, assess how the biochemical, serological, and clinical lab measures have changed with time, and evaluate how previous immune phenotypes predict the health incidences observed in this well-characterized aging cohort.

## Results

### The Milieu Intérieur V3 10-year follow up cohort

**Fig. 1:**
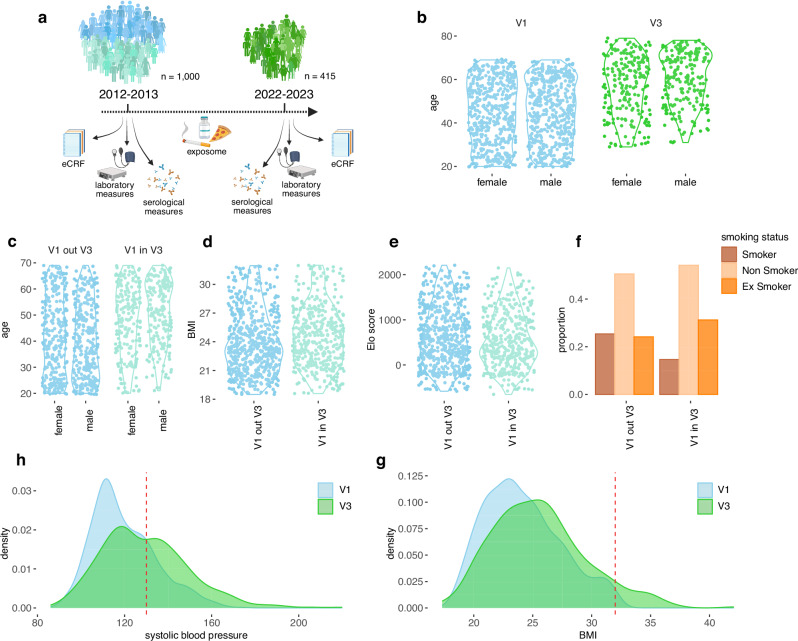
A 10-year follow-up study of the Milieu Intérieur aging cohort. **a** Sampling strategy for the Milieu Intérieur (MI) cohort. **b** Age distribution of MI donors at V1 and V3 visits, according to sex. **c** Age distribution at V1 visit of MI donors who participated (“V1 in V3”) or not (“V1 out V3”) in the follow-up V3 visit, according to sex. **d–f** Distributions of (**d**) BMI, (**e**) ELO score, measuring socio-economic status, and **f** smoking status at V1 visit of MI donors who participated (“V1 in V3”) or not (“V1 out V3”) in the follow-up V3 visit. **h** Systolic blood pressure (in mmHg) density distribution at V1 (*n* = 1000) and V3 visits (*n* = 415). The red dashed line indicates SYSBP = 130 mmHg, the value above which SYSBP is considered high^60^. **g** BMI (in kg/m²) density distribution at V1 and V3 visits. The red dashed line indicates BMI = 32 kg/m², the threshold value used as an inclusion criterion in V1. (**a**) was created with biorender Patin, E. (2026) https://BioRender.com/3vx68kt.

We established the Milieu Intérieur (MI) cohort in 2012–2013 to evaluate how sex, age, and environmental and genetic factors affect inter-individual variation in immune responses. A total of 1,000 healthy donors were recruited in western France, with an equal number of females and males (*n* = 500 of each sex) and participants from five decades of age^14^ (20–69 years, *n* = 200 per decade; Fig. 1a, b). The recruitment was designed to maximize the statistical power to detect effects of sex and age, as well as their interactions, on immunity. In 2021, we initiated a follow-up study of MI participants, referred to as “V3”, 10 years after the first sampling visit “V1” (Fig. 1a), to assess aging effects on immune responses beyond 70 years of age, and to observe whether MI participants have developed disease. We aimed to recruit the maximum number of donors from the original cohort and invited all contactable V1 donors by mail and phone to participate, resulting in the recruitment of 415 participants.

The sex ratio of the follow-up V3 cohort was not different from that of the V1 cohort (*n* = 211 female and 204 and male donors; chi-squared test *P* = 0.68; Fig. 1b). By contrast, there was a bias for age, with older donors more likely to participate in the new study, as shown by significant age differences between V1 donors who participated in the V3 study, compared to those who did not (*t*-test *P* = 9.4 × 10^-19^; Fig. 1c). We next explored potential recruitment bias according to health-related habits or socioeconomic status (SES), using our recently described ELO rating system based on education level, income, and home ownership^24^. We found no significant differences in BMI (Fig. 1d), ELO SES score (Fig. 1e), or smoking status (Fig. 1f) between V1 donors who participated in the V3 study and those who did not (*t*-test *P* > 0.05), while controlling for age. Furthermore, we observed no significant associations between recruitment date (70 dates; *n* = 5.93 samples per date) and age (Kruskal-Wallis test *P*_adj_ = 0.35) or sex (chi-squared test *P*_adj_ = 0.73), nor between clinical investigator (18 investigators; *n* = 23 samples per investigator) and age (*P*_adj_ = 0.24) or sex (*P*_adj_ = 0.10), indicating that age or sex effects on biological measures are not expected to be confounded by potential batch effects.

For the 415 donors, we collected a wide range of demographic data through structured questionnaires, covering health-related habits (physical activity, nutritional habits, sleep quality, etc.) and medical and vaccination records. We also collected 45 biological measurements, including morphological data (e.g., height, weight), general health parameters (e.g., blood pressure, heart rate, temperature), and health biomarkers (e.g., lipid plasma levels, blood cell counts, proteinuria) (Table 1). As expected, we observed that 221 V3 donors no longer met all of the strict inclusion criteria used in V1, suggesting that they had become less healthy with age. For example, participants at V3 have on average higher blood pressure (*t*-test *P* = 2.2 × 10^-−16^; Fig. 1h) and BMI than at the V1 timepoints (*t*-test *P* = 3.4 × 10^-−10^; Fig. 1g). Together, these analyses indicate that the MI follow-up study is well designed to test how aging and age-related common health conditions affect the immune system.

**Table 1:** Sample collections obtained from Milieu Intérieur donors

Data type	Biological measures	Analysis site
Whole blood collection (*n* = 411)		
Complete blood count	RBC, HCT, HGB, MCV, MCH, WBC, NEUTRO, MONO, LYMPHO, EOS, BASO, PLT	Biotrial
Blood electrolytes	Na, K, Ca, P, Cl, HCO3	Biotrial
Liver function tests	HSA, ALP, AST, ALT, GGT, BILI, TPROT	Biotrial
Inflammation	CRP	Biotrial
Renal function tests	BUN, CREAT, UA	Biotrial
Lipids/metabolism	GLUC, TCHOL, LDL, HDL, TRIGLY	Biotrial
Antibody subtype levels: Immunoglobulin electrophoresis	Total IgM, IgG, IgA, IgE serum concentrations	Biotrial
Microbial specific antibody levels: ELISA assays	Hepatitis B (HBs Ag), Hepatitis C (anti-HCV IgG), HIV (anti-HIV IgG, anti-HIV IgM), CMV (anti-CMV IgG), HTLV-1 (anti-HTLV-1 IgG), Influenza	Biotrial
Microbial specific antibody levels: Luminex assays	Immunoglobulin concentrations against 72 microbial antigens	Institut Pasteur
Blood immune cell composition and immunophenotyping: cytometric analysis (Na Heparin tube)	TBD	Institut Pasteur
Functional immune stimulation (Na Heparin syringe): TruCulture tubes for 21 different immune stimuli	TBD	Stored at -80° for later analysis
Genetic variation: DNA sequencing (EDTA tube)	TBD	Stored at -80° for later analysis
Peripheral Blood Mononuclear Cells (PBMCs) (Heparin tube)	TBD	Stored at -80° for later analysis
Plasma (EDTA tube)	TBD	Stored at -80° for later analysis
Urine collection (*n* = 414)		
CLT Biochemistry	proteinuria, glycosuria (dipstick)	Biotrial
CLT Pregnancy test	βHCG concentration (women only)	Biotrial
Fecal sample collection (*n* = 414)		
OMNIgene GUT tube (OMR-200)	TBD	Stored at -80° for later analysis
OMNImet GUT (ME-200)	TBD	Stored at -80° for later analysis
Nasal swabs (*n* = 414)		
Copan FlOQ Swabs (left and right nostril)	TBD	Stored at -80° for later analysis

*CLT* clinical laboratory test, *RBC* red blood cell, *HCT* hematocrit, *HGB* hemoglobin, *MCV* mean corpuscular volume, *MCH* mean corpuscular hemoglobin, *WBC* white blood cell, *NEUTRO* neutrophil, *LYMPHO* lymphocyte, *EOS* eosinophil, *BASO* basophil, *PLT* platelet, *Na* sodium, *K* potassium, *Ca* calcium, *P* phosphorus, *Cl* chloride, *HCO3* bicarbonate, *HSA* human serum albumin, *ALP* alkaline phosphate, *AST* aspartate aminotransferase, *ALT* alanine aminotransferase, *GGT* gamma-glutamyl transpeptidase, *BILI* bilirubin, *TPROT* total protein, *CRP* C-reactive protein, *BUN* blood urea nitrogen, *CREAT* creatinine, *UA* urinalysis, *GLUC* glucose, *TCHOL* total cholesterol, *LDL* low density lipoprotein, *HDL* high density lipoprotein, *TRIGLY* triglycerides, *βHCG* beta-human chorionic gonadotropin. *TBD* stands for ‘to be done’.

### Clinical laboratory measurements in an aging cohort

**Fig. 2:**
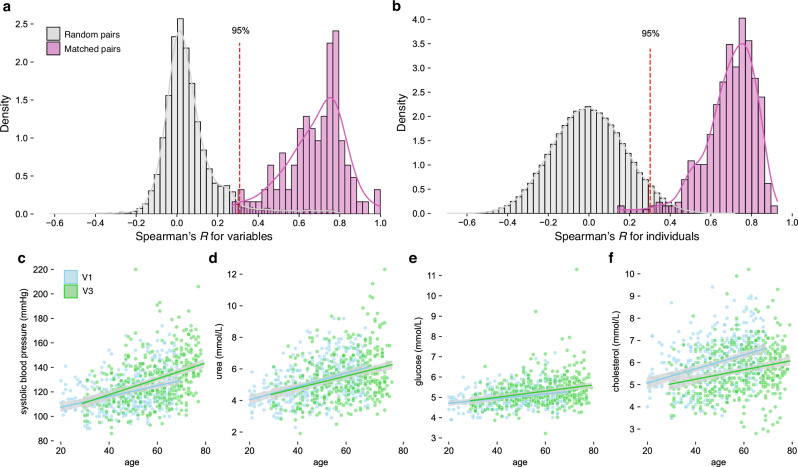
Quality control of the newly collected clinical measurements. **a** Density distributions of paired (purple) and unpaired (gray) Spearman correlation coefficients between clinical measurements, across 411 individuals sampled in V1 and V3. The red dashed line indicates the 95th percentile of the null distribution (gray). Potassium and chlorine concentrations were discarded due to low paired correlations. **b** Density distributions of paired (purple) and unpaired (gray) Spearman correlation coefficients between V1 and V3 individuals, across 107 variables measured in V1 and V3. The red dashed line indicates the 95th percentile of the null distribution (gray). Six individuals were discarded due to low paired correlations. **c–f** Distributions of (**c**) systolic blood pressure, (**d**) urea concentration, (**e**) glycaemia, and (**f**) total cholesterol in V1 (blue, *n* = 1000) and V3 (green, *n* = 405) visits, as a function of age. The variables most associated with age are shown. The solid straight lines indicate the linear regression line. Gray shaded areas indicate the 95% confidence intervals of the regression line.

We evaluated the quality of the newly collected data by computing, for each donor, the correlation between clinical laboratory measurements collected at V1 and V3 visits. To identify potential mismatched pairs of samples, we compared paired correlations to unpaired correlations computed for all donors. We found six donors with unexpectedly low correlations (Spearman’s *r* < 0.30 at FDR = 5%; Fig. 2b and Supplementary Fig. 1), suggesting that these V1 and V3 biological data were not collected from the same donor. Although genetic data will be needed to confirm these observations, conservatively we removed the six donors from all subsequent analyses. We then computed correlations for each biological measurement between 411 V1 and V3 donors with complete data, to identify variables with high measurement error. We found two biological measurements, chlorine and potassium levels, that showed lower V1/V3 correlations than expected (Spearman’s *r* < 0.31 at FDR = 5%; Fig. 2a and Supplementary Fig. 2), relative to other paired correlations. Additionally, we estimated the effect of data sampling batches on the biological measurements, by testing for differences in biological measurements among sampling days or clinical investigators. Out of the 45 variables tested, we found that chlorine (Likelihood ratio test [LRT] *P*_adj_ = 2.2 × 10^-13^; Supplementary Fig. 3), sodium (LRT *P*_adj_ = 1.4 × 10^-8^), and albumin (LRT *P*_adj_ = 6.2 × 10^-5^) levels showed large variation among batches, indicating that these variables are subject to strong technical variation.

We next assessed how age affects these clinical laboratory measurements by leveraging both V1 and V3 data. To discern true age effects from batch effects between V1 and V3 visits, we merged V1 and V3 data and built a model of each biological measurement with age and visit as explanatory variables. Consistent with our previous analyses, we found strong differences between visits for a few variables, including chlorine, bicarbonate, and calcium levels (*t*-test *P*_adj_ < 1.1 × 10^-16^) (Supplementary Fig. 4), indicating high measurement error. Controlling for differences between visits, we found that 34% (16/47) of variables were significantly affected by age (Fig. 2c–f). The biological measurements most associated with age, but not with visit, were systolic blood pressure (β_age_ = 0.54, 95% CI: [0.46 – 0.63]; *t*-test *P*_adj_ = 9.0 × 10^-31^; Fig. 2c), urea levels (β_age_ = 0.040, 95% CI: [0.033 – 0.047]; *t*-test *P*_adj_ = 5.5 × 10^-27^; Fig. 2d) and fasting glycemia (β_age_ = 0.013, 95% CI: [0.010 – 0.016]; *P*_adj_ = 1.1 × 10^-15^; Fig. 2e). Several measurements showed a highly significant age effect and a significant, although low, visit effect, including total cholesterol (β_age_ = 0.026, 95% CI: [0.021 – 0.032]; *t*-test *P*_adj_ = 7.7 × 10^-19^; Fig. 2f) and diastolic blood pressure (β_age_ = 0.022, 95% CI: [0.016 – 0.027]; *t*-test *P*_adj_ = 1.5 × 10^-12^). However, these V1/V3 differences could also be caused by non-linear effects of age. Collectively, the high-quality longitudinal data collected on the MI cohort recapitulates well-known effects of aging on clinical laboratory measurements^27^, while accounting for the batch effects observed between visits.

### Large-scale serotyping and infectious history over a 10-year period

**Fig. 3:**
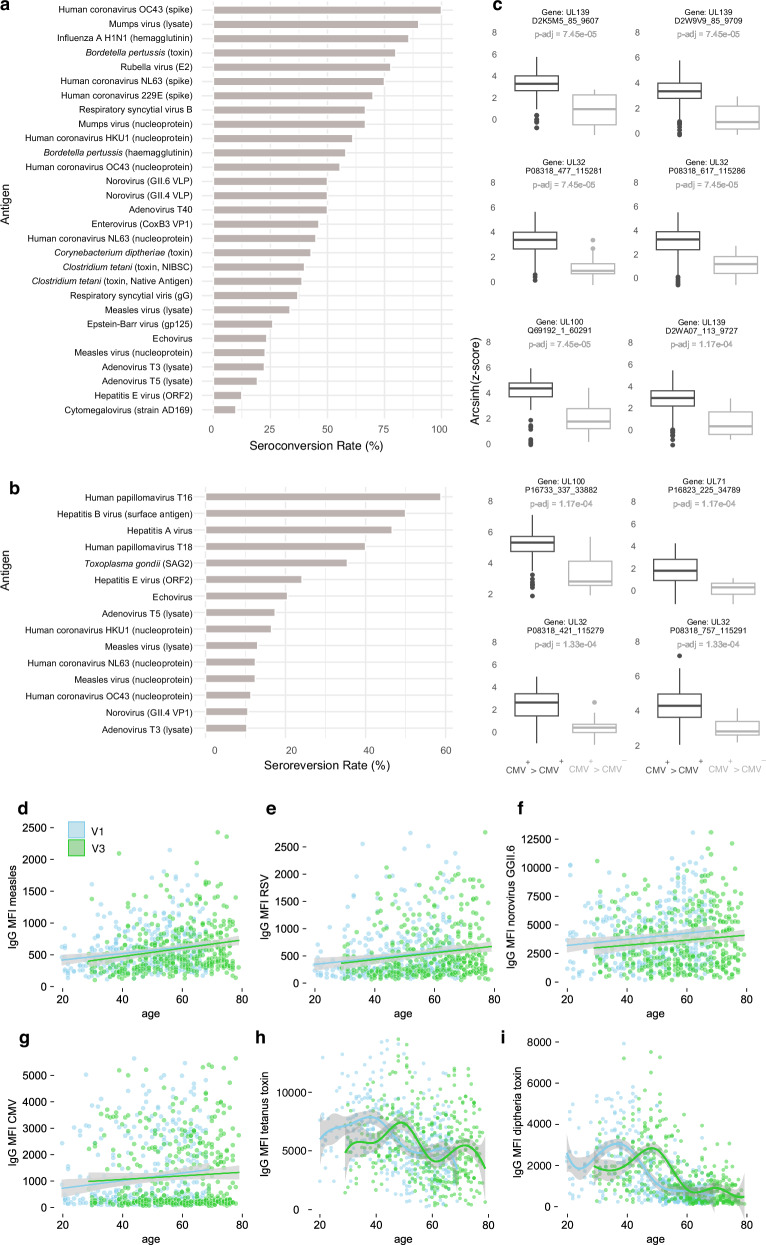
Age-related changes in antibody titers in the Milieu Intérieur aging cohort. **a** Seroconversion and **b** seroreversion rates of MI donors over the follow-up 10-year period, assessed by IgG levels against 45 microbial antigens, measured by multiplex Luminex assays. **c** Differences in antibody reactivity against different CMV peptides, between CMV^+^ V1 donors who remained CMV^+^ or became CMV^–^ at V3 visit. Antibody reactivity was measured by PhIP-seq (VirScan3) in V1 visit. Read counts were normalized and transformed as reported^33^. Boxplots show the median (center line), interquartile range (box limits: 25th–75th percentiles), and whiskers extending to the most extreme data points within 1.5× the interquartile range. CMV+ > CMV + (*n* = 138), CMV+ > CMV + (*n* = 15) **d–i** Distributions of Luminex-based MFIs, in V1 (blue) and V3 (green) visits, measuring IgG titers against (**d**) measles virus, (**e**) respiratory syncytial virus (RSV), (**f**) norovirus, (**g**) cytomegalovirus (CMV), (**h**) *Clostridium tetani* toxin, and (**i**) *Corynebacterium diphtheriae* toxinas as a function of age. (**d-g**) The solid straight lines indicate the linear regression line. (**h,i**) The solid smooth lines indicate the fitted polynomial function with three degrees of freedom. **d–i** Gray shaded areas indicate the 95% confidence intervals of the regression line. (*n* = 405). CMV: cytomegalovirus.

We assessed how antibody responses of MI donors changed over the 10-year period, by measuring, in V1 and V3 plasma samples, immunoglobulin levels against 72 antigens from a diverse array of viruses and bacteria responsible for vaccine-preventable diseases or common infections. Samples from V1 and V3 were randomized and processed with a single reagent lot, to avoid potential measurement biases. Seropositivity could be determined for 45 antigens, based on previously defined cut-offs^28^, and serostatuses were compared between V1 and V3 visits. Restricting analyses to pathogens for which seroconversion rates were significantly higher than 5% (Binomial test *P*_adj_ < 0.05), we identified seroconversions for 29 antigens, indicating repeated or increased exposure with time. Seroconversion rates varied from 9.9% to 100.0%, with an average of 50% per antigen (Fig. 3a). The antigens with the greatest number of seroconversions were, as expected, those related to common infections such as seasonal coronaviruses (NL63, 229E, HKU1, OC43) and influenza A virus (IAV), enteroviruses, and adenoviruses. More surprising was the high seroconversion rate for *Bordetella pertussis* (18/31 = 58.1%; Binomial test *P*_adj_ = 1.8 × 10^-14^) and mumps (9/10 = 90%; Binomial test *P*_adj_ = 8.0 × 10^-10^), given that vaccination is required in children and only recommended in pregnant women and >65-year old individuals in France, suggesting a high incidence of natural infections in the adult population^29,30^.

Conversely, seroreversion rates were significantly higher than 5% for 15 antigens and varied from 10.4% to 58.8%, with an average of 25.3% per antigen (Fig. 3b), implying widespread antibody waning with age and reduced exposure with time. We observed the highest seroreversion rates for vaccine-preventable pathogens, including papillomavirus (HPV16: 10/17 = 58.8%; Binomial test *P*_adj_ = 6.2 × 10^-8^) and hepatitis A, B and E viruses (HAV: 14/30 = 46.7%; HEV: 28/116 = 24.1%), as previously suggested^31,32^. Unexpectedly, we also observed seroreversion for CMV (16/153 = 10.4%; Binomial test *P*_adj_ = 0.041), a herpesvirus that causes persistent latent infection in non-immune compromised donors. To better understand this unexpected result, we leveraged PhIP-seq data collected with VirScan3 in 900 V1 donors^33^, assessing the antibody repertoire against 137 CMV epitopes. We compared anti-CMV antibody repertoires of seropositive V1 donors who became seronegative ten years later, to those of seropositive V1 donors who remained seropositive. We found that the repertoire breadth of seroreverted donors was already lower at V1 visit, relative to that of stable seropositive donors (Wilcoxon test *P*_adj_ < 1.4 × 10^-4^). The CMV epitopes showing the lowest reactivity in seroreverting donors included epitopes from the UL139 ORF, the UL32 gene encoding virion tegument protein (pp150), and the UL100 encoding a viral glycoprotein (Fig. 3c). These results support the view that CMV seroreversion in latently infected individuals is not uncommon^34^ and is caused by the broad decline of anti-CMV antibody titers with age.

To assess age-related changes in quantitative serological measures, which do not require definition of seropositivity thresholds, we next modeled the 72 available antibody levels in V1 and V3 donors as a function of age and visit. We observed an overall increase in anti-measles virus IgGs with age in donors at both time-points (Fig. 3d), suggesting continuous exposure to this virus during adulthood. Similar increases in antibody levels with age were observed for anti-RSV, anti-norovirus and anti-CMV specific IgGs, partly reflecting age-related differences in exposure (Fig. 3e–g)^35,36^. In contrast, antibodies against *Bordetella pertussis* and HPV-16 remained constant with age (Supplementary Fig. 5), implying sustained humoral immunity. Finally, antibodies against *Clostridium tetani* (*t*-test *P* = 1.9 × 10^-14^; Fig. 3h) and *Corynebacterium diphtheriae* (*t*-test *P* = 6.3 × 10^-7^; Fig. 3i) toxins showed a steep decline with age at both time-points, likely reflecting waning immunity in the absence of re-exposure^37^. Interestingly, anti-tetanus and anti-diphtheria antibody levels were the only serological variables showing a significant non-linear effect of age (Fig. 3h, i). Both of these antibody titers peaked in 45-50 year-old V3 donors, perhaps reflecting different lifetime exposures to these antigens through revaccination or infection. Collectively, these results reveal dynamic changes in antibody responses with age in the French population and highlight the potential need of re-vaccination campaigns for such common infections.

### The impact of the COVID-19 pandemic in the MI cohort

**Fig. 4:**
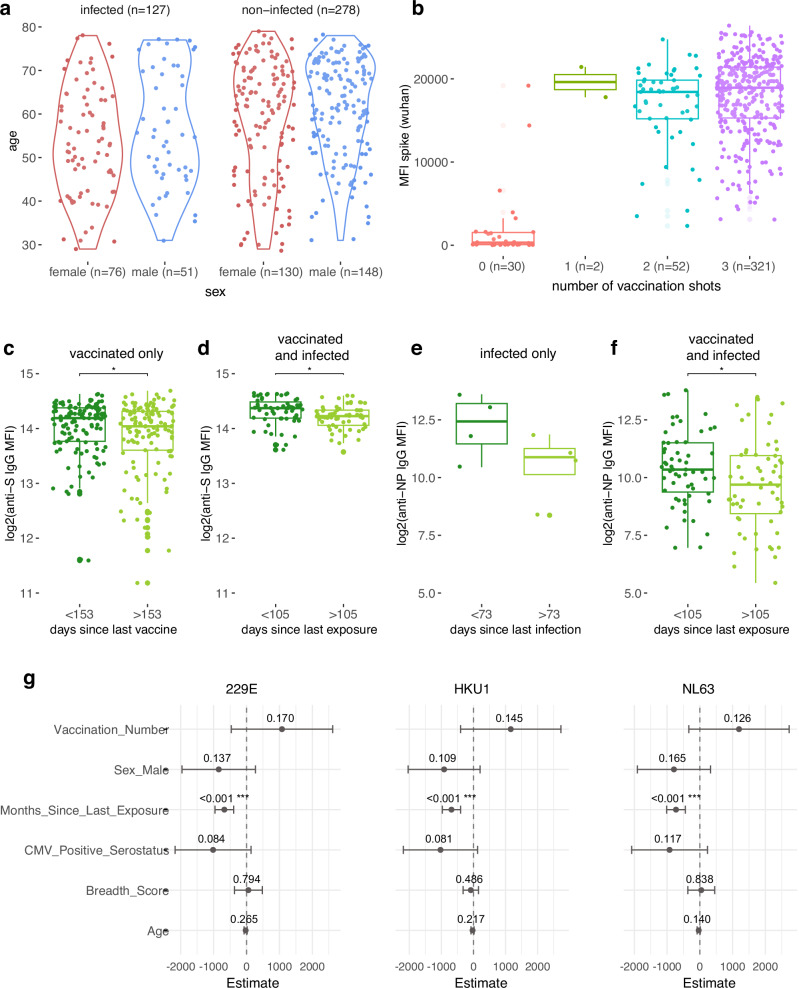
Effects of exposure to seasonal coronaviruses and SARS-CoV-2 antigens on anti-SARS-CoV-2 antibody titers. **a** Age distribution of SARS-CoV-2-infected and non-infected individuals, according to sex. Infection status was confirmed by either PCR or antigenic tests. **b** Anti-Spike IgG titers measured as Mean Fluorescence Intensity (MFI) by Luminex, as a function of the number of COVID-19 vaccine shots received. **c**,**d** Anti-Spike IgG titers in (**c**) vaccinated-only and (**d**) vaccinated and infected individuals, as a function of the number of days since last vaccination or last exposure (vaccination or infection), respectively. (**e-f**) Anti-NP IgG titers in (**e**) infected-only and (**f**) vaccinated and infected individuals, as a function of the number of days since last infection or last exposure, respectively. **c**–**f** Individuals were separated into two equal-sized groups, based on the median number of days since last exposure. * indicates a *P*-value < 0.05 from a multiple regression model adjusted for age and sex. Boxplots show the median (center line), interquartile range (box limits: 25–75th percentiles), and whiskers extending to the most extreme data points within 1.5× the interquartile range. **g** Effects of previous exposure to seasonal coronaviruses on anti-Spike SARS-CoV-2 IgG titers. Effects were estimated in a regression model including, as explanatory variables, age, sex, CMV serostatus, a breadth score measuring antibody reactivity against a seasonal coronavirus (229E, HKU1 or NL63) assessed by VirScan in V1 visit, the number of COVID-19 vaccine doses received, and the number of months since last exposure to SARS-CoV-2 antigens (through either vaccination or infection). Black dots and bars indicate effect size estimates and 95% confidence intervals. (*n* = 405).

Given that the V3 visit occurred in 2022, during the COVID-19 pandemic, the MI follow-up study provided an opportunity to assess responses to acute viral infection with SARS-CoV-2 and to COVID-19 vaccination. Among V3 participants, 31% (127/405) reported a laboratory-confirmed infection with SARS-CoV-2, in agreement with the reported infection rate in France during this period^38^. SARS-CoV-2 infection was associated with younger age (Wald test *P* = 2.2 × 10^-4^) and female sex (Wald test *P* = 0.02; Fig. 4a). Based on reported symptoms and treatments, 55.5% of the SARS-CoV-2 infected donors reported an infection severity index of 3 (Table 2), and only one patient required hospitalization (without respiratory assistance), likely reflecting the overall healthy nature of the cohort. Similarly, the proportion of donors reporting persistent symptoms consistent with long COVID was 15.2% (16/105), lower than rates reported in the general population^39^. Age, sex and six other candidate risk factors were not significantly associated with COVID-19 severity (*t*-test *P*_adj_ > 0.05; Methods), likely due to limited statistical power.

**Table 2:** Clinical manifestations in SARS-CoV-2-infected Milieu Intérieur donors

SARS-CoV-2-infection severity index	Clinical manifestations	Sample size
1	Asymptomatic	14
2	Symptomatic, without treatment	38
3	Symptomatic, with treatment	68
4	Hospitalization without respiratory support	1
5	Hospitalization with respiratory support	0

We then used antibody levels against SARS-CoV-2 nucleoprotein (NP) as a marker of natural infection, by defining the seropositivity cut-off based on V1 pre-pandemic samples. We found that 15.8% (64/405) of participants were positive, of which 19 self-declared no infection. The resulting proportion of asymptomatic infected donors, 29.6% (19/64), is within the range reported in the literature^40^, though a limitation is the poor durability of anti-NP antibodies^41^. Regarding COVID-19 vaccination, most donors reported receiving a vaccination, with only 7% (*n* = 30) not receiving any vaccine, which is lower than the estimated non-vaccination rate in France (13%). Among vaccine recipients, the vast majority (79%) received the recommended three doses, 13% received two doses, and just 0.5% received a single dose (Fig. 4b). Of the first doses received, 70% were with the Pfizer vaccine, 15% with AztraZeneca, 9% with Moderna, and 6% with Johnson and Johnson. As expected, antibodies against the Spike protein (S) of Wuhan SARS-CoV-2 – the target antigen selected in vaccines at this time – were consistently higher with the number of doses received (*t*-test *P* = 1.3 × 10^-44^; Fig. 4b). Both anti-NP and anti-S antibody titers declined several weeks after last exposure, in both vaccinated-only and vaccinated and infected individuals (Fig. 4c-f). Interestingly, vaccinated and infected donors showed boosted vaccine responses – measured by anti-S IgG titers – relative to vaccinated only donors (*t*-test *P* = 1.0 × 10^-5^ and 2.4 × 10^-4^ for recently and formerly exposed donors, respectively; Fig. 4c, d), whereas infected-only donors showed elevated anti-NP antibody titers, compared to vaccinated and infected donors (*t*-test *P* = 0.038 for recently exposed donors; Fig. 4e, f).

We next leveraged the thorough antibody repertoires collected in the Milieu Intérieur cohort at V1 visit to test whether prior exposure and antibody levels against seasonal coronaviruses impacted anti-SARS-CoV-2 humoral responses at V3 visit, an observation previously reported over shorter time frames^42^. We computed a breadth score for NL63, 229E, and HKU1 coronaviruses using the VirScan3 V1 data and tested their association with anti-S antibody titers for the Wuhan SARS-CoV-2 strain at V3 visit in vaccinated participants, while adjusting for age, sex, number of COVID-19 vaccinations and number of days since last exposure (Methods). We found no significant association with breadth scores, nor with reactivity scores for 373 individual NL63 and 229E epitopes, except a weak association with a single HKU1 epitope (*t*-test *P*_adj_ > 0.05; Fig. 4g). In addition, V1 breadth scores for seasonal coronaviruses were not associated with anti-NP IgG titers in SARS-CoV-2-infected donors at V3 visit (*t*-test *P*_adj_ > 0.05). Although our cohort is relatively small for such analysis, these results suggest that previous, long-term exposure to seasonal coronaviruses did not confer humoral protection during the SARS-CoV-2 pandemic.

### Medical events and biological aging over a 10-year period

We examined the medical history of all V3 donors to determine whether they had experienced notable clinical events since their initial recruitment visit (V1). Out of the 405 participants, 133 reported disease occurrences, including cancer, autoimmune disease, cardiovascular disease, acute, chronic or recurrent infection, type 2 diabetes, or severe allergy (Table 3). These donors were classified as ‘diseased’, while the remaining 272 participants were classified as ‘healthy’. We found that the diseased group was significantly older than the control group (odds-ratio [OR] = 1.02, 95% CI: [1.01–1.04]; Wald test *P* = 8.5 × 10^-3^), but did not differ with respect to sex, socio-economic status, smoking status or BMI (Wald test *P* > 0.05), likely reflecting limited statistical power and/or the overall healthy status of Milieu Intérieur participants. We next tested whether V3 clinical laboratory measurements differed between the diseased and control groups, controlling for age and sex. We observed that no individual measure was significantly different between the groups (Wald test *P*_adj_ > 0.05).

**Table 3:** Medical events experienced by Milieu Intérieur donors since initial recruitment

Disease category	Number of recovered participants (*n*)	Details of past/treated diseases (*n*)	Number of diseased participants (*n*)	Details of current diseases (*n*)
Cancers or lymphoma	36	Other (26) Breast (8) Uterus (6) Lung (3) Lymphatic tissue (1)	7	Other (3) Breast (2) Lung (2) Lymphatic tissue (1)
Acquired or congenital immunodeficiencies	7	Immunosuppressive treatment (7)	5	Immunosuppressive treatment (5)
Autoimmune diseases	25	Psoriasis (19) Rheumatoid arthritis (3) Hashimoto (2) Crohn (1)	19	Psoriasis (11) Rheumatoid arthritis (6) Hashimoto (2) Vitiligo (1) Sjogren (1) Crohn (1)
Pulmonary, cardiovascular hepatic or renal diseases	48	Pneumonia (22) Asthma (12) Hypertension (6) Myocardial infarction (3) Chronic bronchitis (3) Pulmonary embolism (2) Cardiac arrhythmia (1)	64	Hypertension (41) Asthma (15) Chronic bronchitis (7) Auricular fibrillation (2) Cardiac arrhythmia (2) Emphysema (1) Pneumonia (1)
Neurological or convulsive problems	4	Epilepsy (3) Peripheral neuropathy (1)	2	Peripheral neuropathy (2) Parkinson (1)
Infectious diseases	-	-	44	Fungal infection (20) Cold syndrome (10) UTI (2) Rhinopharyngitis (2) Sinusitis (2) Chickenpox (2) Whooping cough (2) Bronchitis (1) HPV (1) Lyme disease (1) Tuberculosis (1) Conjunctivitis (1) Otitis (1)
Chronic bone diseases	1	Osteoporosis (1)	14	Osteoporosis (14)
Significant coagulation problems	8	Anti-coagulant treatment (8)	1	Anti-coagulant treatment (1)
Severe acute/chronic allergies	7	Bite allergy (3) Severe allergy (2) Food allergy (1) Dermatitis (1)	10	Severe allergy (5) Dermatitis (3) Bite allergy (2) Food allergy (1)

**Fig. 5:**
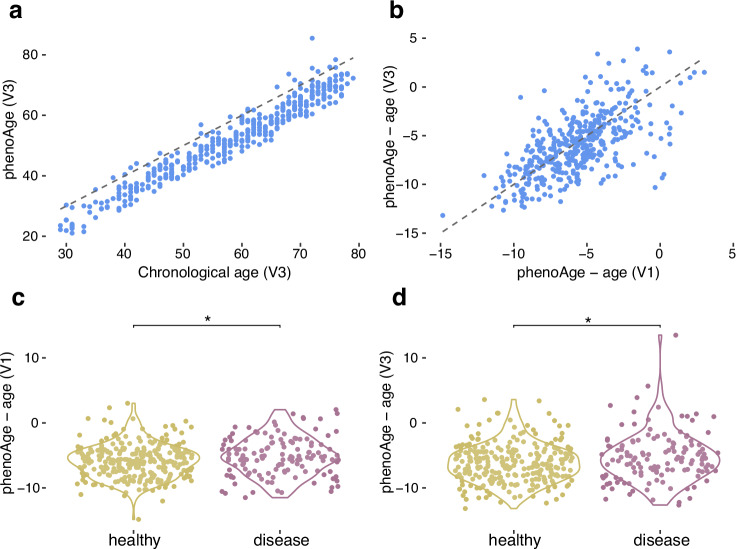
Disease status and phenotypic age in the Milieu Intérieur cohort. **a** Chronological age compared with phenotypic age in V3 visit. **b** Phenotypic age acceleration in V1 visit compared with phenotypic age acceleration in V3 visit. Phenotypic age acceleration refers to the difference between phenotypic and chronological ages. **a**, **b** The gray dashed lines indicate the identity line. **c**, **d** Phenotypic age acceleration estimated in (**c**) V1 and (**d**) V3 visits, as a function of disease status in V3 visit. * indicates *P*-value < 0.05 from a regression model of disease status with phenotypic age as explanatory variable and chronological age and sex as covariates. (*n* = 405, disease=133, healthy=272).

We searched for clinical laboratory biomarkers measured during the V1 visit that could predict disease development 10 years later. We found that none of the individual biological and serological variables in V1 data differed significantly between healthy and diseased V3 donors (Wald test *P*_adj_ > 0.05). We then tested association with the PhenoAge score^43^, a predictor of morbidity and mortality built upon chronological age and measured levels of albumin (*β*_age_ = –0.040; *t*-test *P*_adj_ = 2.6 × 10^-10^), creatinine (*β*_age_ = 0.125; *t*-test *P*_adj_ = 5.8 × 10^-4^), glucose (*β*_age_ = 0.013; *t*-test *P*_adj_ = 1.2 × 10^-15^), alkaline phosphatase (*β*_age_ = 0.381; *t*-test *P*_adj_ = 8.2 × 10^-11^), C-reactive protein (*β*_age_ = 0.005; *t*-test *P*_adj_ = 0.567), the proportions of leukocytes (*β*_age_ = –0.015; *t*-test *P*_adj_ = 1.1 × 10^-3^) and lymphocytes (*β*_age_ = –0.007; *t*-test *P*_adj_ = 9.6 × 10^-5^) and mean corpuscular volume (*β*_age_ = 0.049; *t*-test *P*_adj_ = 2.7 × 10^-6^). Expectedly, PhenoAge was strongly correlated with chronological age in both V1 and V3 cohorts (Pearson’s *R*² = 0.98 and 0.97, respectively; Fig. 5a). Biological age acceleration, defined as the difference between phenotypic and chronological age, was correlated between V1 and V3 time-points (Pearson’s *R*² = 0.38; Fig. 5b), indicating that accelerated aging is consistent over a 10-year interval. Interestingly, we observed that PhenoAge of V1 donors was significantly higher in diseased V3 donors relative to healthy V3 donors, when controlling for chronological age (OR = 1.13, 95% CI: [1.04 – 1.22]; Wald test *P* = 1.8 × 10^-3^) (Fig. 5c). Differences were also significant when comparing the V3 PhenoAge score between healthy and diseased groups (OR = 1.11, 95% CI: [1.04 – 1.19]; Wald test *P* = 2.3 × 10^-3^; Fig. 5d). Together, these results indicate that an aggregated score measuring accelerated biological aging is associated with disease development in the Milieu Intérieur cohort.

### Immune response differences in donors who later developed disease

**Fig. 6:**
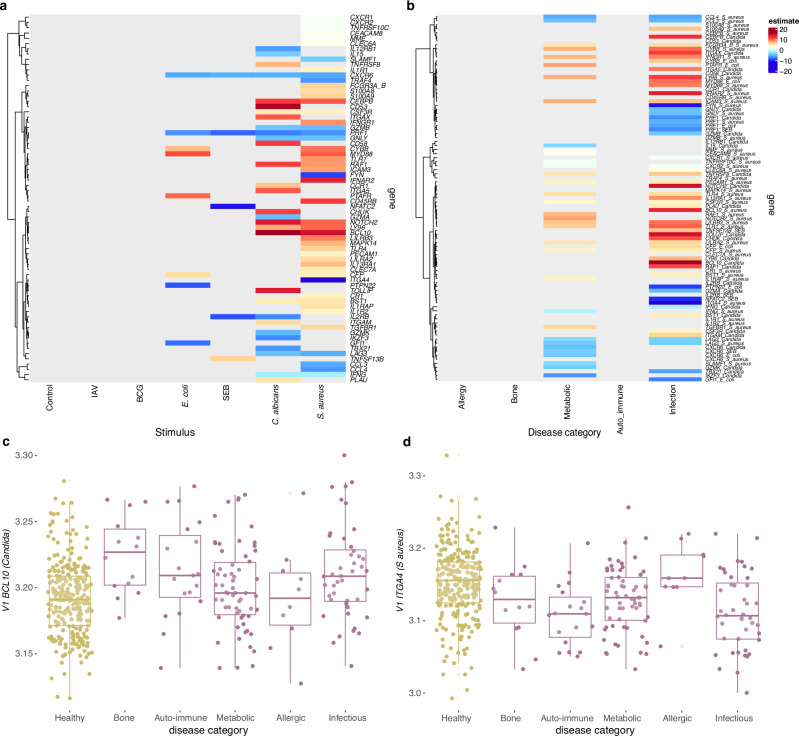
Associations between disease status and induced expression of immune genes measured ten years previously. **a** Differences in immune gene expression measured in V1 visit between healthy and diseased MI donors in V3 visit. Gene expression was measured by NanoString for 560 immunity-related genes, before or after whole blood stimulation by six immune stimuli. **b** Differences in immune gene expression measured in V1 visit between healthy and diseased MI donors in V3 visit, according to disease categories. **a**,**b** Differences were estimated as the effect size of gene expression on disease status, adjusting for age and sex. Only FDR corrected significant genes are shown. **c**,**d** Gene expression levels of **c** *BCL10* and **d** *ITGA4* upon whole blood stimulation by *C. albicans* and *S. aureus*, respectively, according to disease categories. (*n* = 405, disease=133, healthy=272). Boxplots show the median (center line), interquartile range (box limits: 25th–75th percentiles), and whiskers extending to the most extreme data points within 1.5× the interquartile range.

Blood cell composition was extensively characterized by standardized flow cytometry in the Milieu Intérieur V1 cohort, enabling us to test if immune cell phenotpyes at V1 were associated with disease development later in life. The immunophenotypes included 76 absolute counts of both innate and adaptive immune cells, 87 expression levels of cell-surface markers (quantified as mean fluorescence intensity), and 3 cell ratios^15^. We found no significant differences (Wald test FDR > 0.05) in immunophenotypes between donors who later developed disease and those who remained healthy according to our original criteria. We next hypothesized that altered functional immune responses to pathogens, rather than those in steady state, may be a better indicator of later disease development. We thus tested by logistic regression if the expression of 560 immune genes, measured at V1 visit after whole blood stimulation by six different microbes or their components^17^, differed between diseased and healthy donors at V3 visit. We found significant associations between later disease status and the induced mRNA levels of 91 genes, primarily after stimulation by *Candida albicans* and *Staphylococcus aureus* at V1 (Wald test FDR < 0.05; Fig. 6a). Notably, no associations were detected in the non-stimulated condition, supporting the view that transcriptional activity under homeostasis is less informative of subsequent health conditions than functional immune responses. Interestingly, pattern recognition receptor and toll-like receptor pathways were enriched in the disease-associated genes (chi-squared test *P*_adj_ = 0.005), suggesting that weakened innate immune responses may increase overall risk to later develop disease.

In this initial analysis, we merged donors with heterogenous pathological conditions into a single disease group, to gain statistical power. However, these diseases affect different organs and have distinct etiologies, suggesting that the observed associations with gene expression may be driven by specific pathologies. After excluding categories with very low incidence in our cohort (i.e., cancer, neurological disease, coagulation deficiency or immune deficiency), we sub-classified diseased patients into 5 categories, which included acute/chronic infections (e.g., fungal infection, cold syndrome), acute/chronic allergies (e.g., dermatitis), auto-immune diseases (e.g., psoriasis, rheumatoid arthritis), skeletal diseases (osteoporosis) and pulmonary and cardiovascular diseases (e.g., hypertension, asthma) (Table 3). Interestingly, we observed that expression of the 91 disease-associated genes differed mainly in patients affected by infectious and metabolic conditions (Wald test FDR < 0.05; Fig. 6b). Notably, *BCL10* mRNA levels upon *C. albicans* stimulation were markedly higher in patients with infections, relative to healthy controls (Fig. 6c). BCL10 plays a critical role in host defense through NF-κB signaling, and its impaired expression predisposes to recurrent infection^44,45^. Conversely, *S. aureus*-induced expression of *ITGA4* gene was lower in patients from most sub-categories, relative to healthy controls (Fig. 6d). This gene encodes a subunit of integrin α4β1 (VLA-4), which recruits lymphocytes to inflamed tissues^46^ and is a target of anti-inflammatory treatments^47^. Collectively, these results reveal that altered transcriptional responses of immune genes to microbial stimulation could be used as predictive biomarkers of later disease development.

## Discussion

We present here a longitudinal study of the Milieu Intérieur cohort, comprising 415 of the original 1000 donors sampled a decade ago. In this new V3 study, expected signs of biological aging were evident, alongside the emergence of diseases within this initially healthy population. We leveraged detailed immunophenotyping of the cohort from the initial visit to identify immune parameters that may confer a heightened risk to develop disease later in life. Interestingly, our analyses revealed clear differences in transcriptional responses to immune stimulation between individuals who went on to develop infectious or metabolic disease during the following ten years, relative to those who remained healthy (Fig. 6). Notably, such immune differences were not observed when comparing baseline gene expression levels or cellular immune phenotypes, highlighting the clinical relevance of whole-blood stimulation assays for immune function. Our findings strongly suggest that altered immune states in apparently healthy individuals—assessed through functional responses to microbial challenges—can serve as promising predictors of age-related conditions. Interestingly, induced responses to certain microbes (e.g., *S. aureus* and *C albicans*) showed more gene expression changes associated with disease as compared to other microbes (e.g., Influenza and BCG). This raises intriguing hypotheses such as a greater implication of commensal versus non-commensal microbes in shaping such responses, or alternatively a potential influence of confounding factors (e.g., nonlinear age effects for influenza responses) that may not be captured. Future analyses integrating immune pathway specific responses may provide greater understanding of the underlying mechanisms. In addition, a combined biological aging score (PhenoAge)^43^ was significantly higher in individuals who develop disease (Fig. 5), further highlighting the potential of biomarker-based clinical scores for accessible health monitoring^48^. Nonetheless, these observations now require validation in larger longitudinal cohorts, as the heterogeneity and relative low frequency of diseases observed in the MI cohort pose challenges for investigating specific pathologies in isolation.

A major challenge in longitudinal studies is managing technical variability in assay measurements over time. While batch effects can often be addressed using statistical methods^49^, they become more problematic when fully confounded with group structure, as is possible in studies conducted years apart. With this in mind, and to ensure the high-quality nature of our cohort and data set, we assessed the correlation of biological measurements between the two time-points for each donor and found strong correlations in the vast majority of cases. For donors with lower-than-expected correlations, potential explanations include individual mismatches (which appear unlikely given consistent demographic data) or major physiological changes over the decade. Although future genetic analyses will provide definitive clarification, we have conservatively excluded these donors from downstream analyses. Reassuringly, comparisons of clinical laboratory measures between visits also showed strong correlations (Fig. 2), further supporting the quality of the data collected.

The timing of our follow-up study, which began soon after the beginning of the COVID-19 pandemic, offerred a unique opportunity to explore factors influencing individual variability in responses to SARS-CoV-2 infection and vaccination. Based upon medical records and serological data, we were able to differentiate infected from non-infected donors, and symptomatic from asymptomatic cases, a group that is challenging to identify in conventional studies^40^. High-throughput serotyping revealed that prior exposure to seasonal coronaviruses does not correlate with milder COVID-19 symptoms or enhanced vaccine response (Fig. 4). These findings support the limited cross-reactivity of anti-SARS-CoV antibodies with SARS-CoV-2, in line with another longitudinal study^50^. Additionally, we found that individuals who were naturally-infected with SARS-CoV-2 mounted stronger vaccine responses than those who were vaccinated but never infected, reinforcing prior evidence that natural infection enhances subsequent vaccine responses^51^. Ongoing studies aim to further characterize SARS-CoV-2-specific systemic and mucosal immunity, alongside non-specific innate immune pathways, to provide deeper insight into the cellular and molecular mechanisms underlying these observations.

Besides COVID-19, our data reveal that new infections were common over the 10-year period, with particularly high seroconversion rates for common infections such as enterovirus and seasonal coronaviruses. More surprisingly, longitudinal serological data also revealed high seroreversion rates and declining antibody levels with age for several pathogens of public health relevance, including papillomavirus, hepatitis viruses, diphtheria, and CMV (Fig. 3). These results highlight that waning immunity is a common phenomenon and emphasize the importance of mantaining vaccine booster campaigns in aging populations. In summary, this cohort offers a valuable resource for studying immune aging, providing a proof-of-concept that functional immune signatures can predict deviation from a healthy status. Future studies should now elucidate the specific immune mechanisms underlying altered responses in pathological conditions.

## Methods

### Clinical protocol and implementation

Our research complies with all relevant ethical regulations as described below. The 10-year follow-up Milieu Intérieur study, referred to as MI visit 3 (“V3”), was approved by the Comité de Protection des Personnes — Nord Ouest III (Committee for the protection of persons) on 27th January 2022, and by the French Agence nationale de sécurité du médicament (ANSM) on 30th November 2021. The study was sponsored by the Institut Pasteur (ID-RCB Number: 2021-A02621-40) and conducted as a single-center study without any investigational product. The protocol is registered at ClinicalTrials.gov (study# NCT05381857). As this study was designed to be a 10-year follow-up of the original Milieu Intérieur “V1” cohort (NCT01699893 and NCT03905993), the major inclusion criterion was previous inclusion in the V1 study. In addition, donors were required to give written informed consent, and be affiliated to the French social security system. Non-inclusion criteria for these pre-screened individuals were restricted to ongoing pregnancy, inability to provide informed consent, or specific reasons requiring legal protective measures. No donors were excluded for any of these non-inclusion criteria. In total, 415 subjects from the previous Milieu Intérieur cohort were included. As self-reported Metropolitan French origin for three generations was an inclusion criterion of the original study, the majority of the donors are of Western European ancestry, which was confirmed through genomic analyses^15^. All subjects received compensation for their participation.

### Donor recruitment

As for the original Milieu Intérieur study, donors were recruited at Biotrial, Rennes, France (Contract Research Organization, SIREN: 351 643 523). Donors were first informed of the study through an information leaflet, which invited them to participate to an online webinar. During the webinar, results from the first study were presented and participants had opportunities to ask questions to scientists from the consortium (https://www.milieuinterieur.fr/en/contact-us/webinar/). In the second stage, donors were contacted directly through invitation letters, emails, and telephone calls.

### Biological sampling

Donors were interviewed and sampled during a single visit at Biotrial (Rennes, France) between the period of 14^th^ March 2022 and 12^th^ October 2022. After completing a REDCap-based healthcare and lifestyle questionnaire (Supplementary Text) with a medical practitioner, all donors underwent a complete medical check, as originally described^14^, and provided 100 mL of blood and two nasal swab samples (Table 1). Fecal samples were collected at home by the donors in the days prior to the medical visit in Omnigene Gut OMR-200 tubes, which were frozen at -80 °C upon reception at the clinic. Whole blood was used for routine clinical and biochemical laboratory testing (21 mL) (Table 1), as well as immediate incubation in TruCulture whole blood assays for 21 different immune ligands (25 mL total, 1 mL per stimulus), and 54 mL was transported on the same day under temperature monitoring to the Institut Pasteur, Paris for flow cytometry analysis and peripheral blood mononuclear cells (PBMCs), plasma, and DNA isolation.

### Luminex-based serotyping

Serum samples were tested for antibodies to a broad panel of common respiratory pathogens and routine vaccine-preventable diseases using bead-based multiplex assays. A 61-plex assay was developed to include antigens for adenovirus, cytomegalovirus (CMV), Epstein-Barr virus (EBV), echovirus, enterovirus CoxB3, Hepatitis A virus (HAV), Hepatitis B virus (HBV), Hepatitis C virus (HCV), measles, Varicella-Zoster virus (VZV), mumps, rubella, norovirus, respiratory syncytial virus (RSV), rhinovirus, rotavirus, human papillomavirus, and influenza A virus (IAV). In parallel, a 24-plex assay was developed for SARS-CoV-2 antigens and human seasonal coronaviruses including 229E, NL63, OC43, and HKU1. Development of these assays, including the antigens used, are described elsewhere^28^. The proteins used were either purchased from Native Antigen (Oxford, UK), ProSpec-Tany Techno Gene (Israel), or Ray Biotech (Georgia, US). Samples were run at a final dilution of 1:200. Plates were read using a Luminex IntelliFlex system, and the median fluorescence intensity (MFI) was used for data analysis. Samples from V1 and V3 visits were randomized in the same batch. We discarded antibody levels for which seropositivity could not be defined unambiguously, showing evidence of cross-reactivity or poor reproducibility^28^. After quality control assessment, 72 serological measures were retained for subsequent analysis.

### Clinical data collection

Clinical data were collected and managed using REDCap electronic data capture tools^25^ (Research Electronic Data Capture v11.4.4) hosted at Institut Pasteur (Paris, France). REDCap is a secure, web-based software platform designed to support data capture for research studies, providing (i) an intuitive interface for validated data capture; (ii) audit trails for tracking data manipulation and export procedures; (iii) automated export procedures for seamless data downloads to common statistical packages; and (iv) procedures for data integration and interoperability with external sources^52^. Data were collected by Biotrial between 14^th^ March 2022 and 28^th^ October 2022. The electronic clinical report form (eCRF) included a total of 1,050 questions and was divided across ten thematic forms: general information, medical history, cancers, vaccinations, infectious diseases, COVID-19, other information, food habits, biological samples, and adverse events. The questionnaire, written in French, is provided as Supplementary Text.

### Clinical data cleaning

After clinical data collection, each form was exported from REDCap separately and imported in the R software (v. 4.3.0). Data cleaning comprised various steps: standardization of the variable names, formatting of the date variables to YYYY-MM-DD ISO-8601 format, English translation and uniformization of the ‘free text’ variables with regular expressions. Several quality controls were performed; including the detection of age and sex discrepancies, and the detection of outlier values in the clinical data, defined as values outside of expected boundaries provided by Biotrial. Each discrepancy/outlier value was reported to Biotrial and corrected directly in REDCap, whenever possible.

### Data processing

Two donors presented with poor venous capital and one showed evidence for hemolysis, which is known to impact the quality of laboratory measurements. These donors were excluded from biological analyses. One additional donor was excluded because of ongoing breast-feeding. In total, 411 out of 415 donors were kept for biological analysis, with 415 used for questionnaire-based analysis. For all V1 and V3 biological and serological quantitative measurements, only three data points were missing and were imputed with the K-Nearest-Neighbors (K = 5) imputer (scikit-learn package v1.4.1)^26^. Eleven outlier values, defined as physiologically unlikely data points out of the interval bounded by the first quartile – 4.5 × interquartile range (IQR) and the third quartile + 4.5 × IQR, were replaced by the median of the distribution. For specific analyses, variables were either scaled between 0 and 1 by min-max scaling or converted into *Z*-scores by standardization, to facilitate comparisons across variables. All the processing steps were performed in Python 3.10.4 with packages numpy^53^ 1.26.4, pandas 2.2.1, scikit-learn 1.4.1, in a Jupyter-lab 3.4.2 web-based notebook.

### Immunological data collected at V1 visit (2012-2013)

Immunological data sets that were collected from the original V1 study included 169 circulating blood immune cellular phenotypes assessed by standardized, multi-parameter flow cytometry^15^; gene expression measures of 560 immune genes assessed by Nanostring before and after TruCulture whole blood stimulation with three bacteria (*Escherichia coli*, *Staphylococcus aureus*, and Bacillus Calmette–Guérin (BCG)), a fungus (*Candida albicans*), a live virus (IAV) and a superantigen, staphylococcal enterotoxin B (SEB)^17^; and antibody reactivity scores assessed by phage-display immunoprecipitation sequencing (PhIP-seq) for 2,608 viral peptides included in the VirScan3 assay, as well as breadth scores for 133 viruses computed with AVARDA^54^ from the VirScan3 data^33^.

### Statistical analyses and data visualization

To identify clinical measurements showing poor reproducibility between visits (Fig. 2a), we computed Spearman correlation coefficients for every possible pair composed of a variable measured in V1 and a variable measured in V3, across the 411 individuals recruited in V1 and V3 visits. Matched and unmatched pairs were defined as pairs comparing the same variable or different variables, respectively. We excluded two measurements, Potassium (ρ = 0.27) and Chlorine (ρ = 0.30), for which the matched pair showed a correlation coefficient lower than 0.31, which was the 95% percentile of the distribution of correlation coefficients for unmatched pairs.

To identify samples showing poor reproducibility between visits (Fig. 2b), we computed Spearman correlation coefficients for every possible pair composed of one V1 sample and one V3 sample, across the 105 standardized quantitative measurements obtained. We defined a mismatched pair as a pair of V1 and V3 samples with the same identifier showing a correlation coefficient lower than 0.30, which was the 95% percentile of the distribution of correlation coefficients for unmatched pairs. We excluded 6 donors with correlation coefficients ranging from 0.14 to 0.29.

Statistical tests and multiple regression analyses were conducted in Python 3.10.4, with packages scipy 1.12.0, statsmodels 0.14.0. R software 4.3.0 was also used for Generalized Linear Models (GLM) and Generalized Linear Mixed Models (GLMM) with R packages lmerTest^55^ 3.1-3 and lme4^56^ 1.1-35.1. To assess non-linear effects of age, spline regressions and Likelihood-ratio tests were performed with R packages splines 4.3.0 and lmtest 0.9-40. Tests were corrected for multiple testing with the Bonferroni correction. Data visualization was performed using Python package Matplotlib 3.8.0 and Seaborn 0.13.2, or R package ggplot2 3.4.3 and Patchwork 1.2.0. A conda environment recipe file in yaml format with the specified package versions is available upon request, to rebuild the environment and reproduce the results. When the same hypothesis was tested multiple times, *P-*values were corrected for multiple testing by computing False Discovery Rate (FDR) adjusted *p*-values, with FDR < 0.05 considered significant.

Candidate risk factors for SARS-CoV-2 infection and COVID-19 severity included age, sex, smoking status, BMI, abdominal circumference, blood pressure, heart rate and C-reactive protein concentration^57^. The SARS-CoV-2 infection severity score was computed as previously described^58^, based on self-declared symptoms, treatments and hospitalization.

PhenoAge was computed with the R package BioAge^59^ 0.1.0. As the red cell distribution width (RDW) variable was missing from the MI data, we trained a new PhenoAge model on the NHANES III data without the RDW predictor. The metabolic score, which varies from 0 to 1, was computed for each donor by incrementing the score by 1 when: abdominal circumference > 94 cm or > 80 cm in men or women, respectively; systolic blood pressure ≥ 130 mmHg or diastolic blood pressure ≥ 85 mmHg; triglyceride levels ≥ 1.7 mM; HDL levels <1 mM or <1.3 mM in men or women, respectively; glucose concentration ≥ 6.1 mM^60^. BioRender was used for Fig. 1a visualization.

### Reporting summary

Further information on research design is available in the Nature Portfolio Reporting Summary linked to this article.

## Supplementary information

Supplementary Information

Reporting Summary

Transparent Peer Review file

## Data Availability

All the Milieu Intérieur datasets used in this study, including demographic data, clinical laboratory measures, and serological values collected in V1 and V3 visits, can be accessed from the Institut Pasteur Owey data repository (https://dataset.owey.io). Requests for data and/or sample access can be submitted by filling an online form (https://redcap.pasteur.fr/surveys/?s = ND8TP8MDD3), which will be automatically received by the Milieu Interieur Data Access committee (DAC). The DAC is required to inform all the research participants of the data access request and grants data access if the request is consistent with the informed consent signed by the participants. In particular, research on Milieu Intérieur datasets is restricted to research on the genetic and environmental determinants of human variation in immune responses. Data access is typically granted two months after request submission.
