# Supplementary material for: An integrative approach for studying immunological variation in an aging population – The Milieu Intérieur follow-up study
Source: Nat Commun. 2026 May 22;17:6078. doi: 10.1038/s41467-026-72910-x (PMC13354573; doi:10.1038/s41467-026-72910-x)
Supplement: Supplementary file 1 — Supplementary Information [file 41467_2026_72910_MOESM1_ESM.pdf]

Supplementary Information

Milieu Intérieur Consortium¶

Laurent Abel[10],[11], Andres Alcover[12], Hugues Aschard[13], Philippe Bousso[14], Nollaig Bourke[15], Petter Brodin[16,17], Pierre Bruhns[18], Nadine Cerf-Bensussan[1]9, Ana Cumano[20], Christophe D'Enfert[21], Caroline Demangel[22], Ludovic Deriano[23], Marie-Agnès Dillies[24], James Di Santo[25], Gérard Eberl[26], Jost Enninga[27], Jacques Fellay[28,29], Ivo Gomperts-Boneca[30], Milena Hasan[4], Gunilla Karlsson Hedestam[31], Serge Hercberg[32,33], Molly A Ingersoll[34], Olivier Lantz[35,36], Rose Anne Kenny[15,37], Mickaël Ménager[38], Frédérique Michel[39], Hugo Mouquet[40], Cliona O'Farrelly[41,42], Etienne Patin[1], Antonio Rausell[43,44], Frédéric Rieux-Laucat[45], Lars Rogge[46], Magnus Fontes[47], Anavaj Sakuntabhai[48,49], Olivier Schwartz[50], Benno Schwikowski[51], Spencer Shorte[52], Frédéric Tangy[53], Antoine Toubert[54], Mathilde Touvier[32,33], Marie-Noëlle Ungeheuer[55], Christophe Zimmer[56,57,58], Matthew L. Albert[59], Darragh Duffy[3,4§], Lluis Quintana-Murci[1,9§]

§ co-coordinators of the Milieu Intérieur Consortium

Additional information can be found at:

https://www.milieuinterieur.fr/en/

[1]Human Evolutionary Genetics Unit, Institut Pasteur, Université Paris Cité, CNRS UMR2000, Paris 75015, France

[3]Translational Immunology Unit, Department of Immunology, Institut Pasteur, Université Paris Cité, Paris 75015, France

[4]Single Cell Biomarkers UTechS, Institut Pasteur, Université Paris Cité, Paris 75015, France

[9]Chair Human Genomics and Evolution, Collège de France, Paris 75005, France

[10] Laboratory of Human Genetics of Infectious Diseases, Necker Branch, Inserm U1163, Necker Hospital for Sick Children, Paris, France

[11] St. Giles Laboratory of Human Genetics of Infectious Diseases, Rockefeller Branch, Rockefeller University, New York City, NY, USA

[12] Unité Biologie Cellulaire des Lymphocytes, Institut Pasteur, Université Paris Cité, INSERM U1224, Paris, France

[13] Statistical Genetics Unit, Institut Pasteur, Université Paris Cité, Paris, France

[14] Dynamics of Immune Responses Unit, Institut Pasteur, Université Paris Cité, Paris, France

[15] Trinity Translational Medicine Institute, Trinity College Dublin, Ireland

[16] Department of Women's and Children's Health, Karolinska Institutet, Sweden

[17] Department of Immunology and Inflammation, Imperial College London, UK

[18] Antibodies in Therapy and Pathology Unit, Institut Pasteur, Université Paris Cité, Paris, France

[19] Laboratory of Intestinal Immunity, Imagine Institute, Université Paris Cité, Paris, France

[20] Unit of Lymphocytes and Immunity, Institut Pasteur, Université Paris Cité, Paris, France

[21] Unité Biologie et Pathogénicité Fongiques, Institut Pasteur, Université Paris Cité, Paris, France

[22] Immunobiology and Therapy Unit, Institut Pasteur, Université Paris Cité, Paris, France

[23] Genome Integrity, Immunity and Cancer Unit, Institut Pasteur, Université Paris Cité, Paris, France

[24] Bioinformatics and Biostatistics Hub, Institut Pasteur, Université Paris Cité, Paris, France

[25] Innate Immunity Unit, Institut Pasteur, Université Paris Cité, Inserm U1223, Paris, France

[26] Microenvironment and Immunity Unit, Institut Pasteur, Université Paris Cité, Paris, France

[27] Dynamics of Host-Pathogen Interactions Unit, Institut Pasteur, Université Paris Cité, Paris, France
[28] EPFL, Lausanne, Switzerland
[29] Lausanne University Hospital, Switzerland
[30] Bacterial Cell Wall Genetics Unit, Institut Pasteur, Université Paris Cité, Paris, France
[4] Single Cell Biomarkers Unit, Institut Pasteur, Université Paris Cité, Paris, France
[31] Karolinska Institutet, Stockholm, Sweden
[32] CRESS-EREN, INSERM, Bobigny, France
[33] NACRe Network, France
[34] Mucosal Immunology Unit, Institut Pasteur / Institut Cochin, Université Paris Cité, Paris, France
[35] Institut Curie, INSERM U932, Paris, France
[36] CIC-BT1428, Institut Curie, Paris, France
[37] Mercer's Institute for Successful Ageing, Dublin, Ireland
[38] Imagine Institute, Single-Cell Lab, Paris, France
[39] Cytokine Signaling Unit, Institut Pasteur, Université Paris Cité, Paris, France
[40] Humoral Immunology Unit, Institut Pasteur, Université Paris Cité, Paris, France
[41] School of Biochemistry and Immunology, Trinity College Dublin, Ireland
[42] School of Medicine, Trinity College Dublin, Ireland
[1] Human Evolutionary Genetics Unit, Institut Pasteur, Université Paris Cité, Paris, France
[43] Computational Systems Biomedicine Lab, Institut Pasteur, Université Paris Cité, Paris, France
[44] Institut Pasteur–Oncovita Joint Lab, Paris, France
[45] Clinical Genetics Unit, Necker Hospital, Paris, France
[46] Immunoregulation Unit, Institut Pasteur, Université Paris Cité, Paris, France
[47] Institut Roche, Boulogne-Billancourt, France
[48] Ecology and Emergence of Pathogens Unit, Institut Pasteur, Université Paris Cité, Paris, France
[49] University of Tokyo (IMSUT), Japan
[50] Virus and Immunity Unit, Institut Pasteur, Paris, Université Paris Cité, France
[51] Systems Biology Unit, Institut Pasteur, Université Paris Cité, Paris, France
[52] UTechS Platform, Institut Pasteur, Université Paris Cité, Paris, France
[53] Viral Genetics Unit, Institut Pasteur, Université Paris Cité, Paris, France
[54] Institut de Recherche Saint Louis, Paris, France
[55] ICAReB Biobank, Institut Pasteur, Université Paris Cité, Paris, France
[56] Imaging and Modeling Unit, Institut Pasteur, Université Paris Cité, Paris, France
[57] University of Würzburg, Bioimaging Center, Germany
[58] University of Würzburg, AI & Data Science Center, Germany
[59] Octant Inc., Emeryville, CA, USA

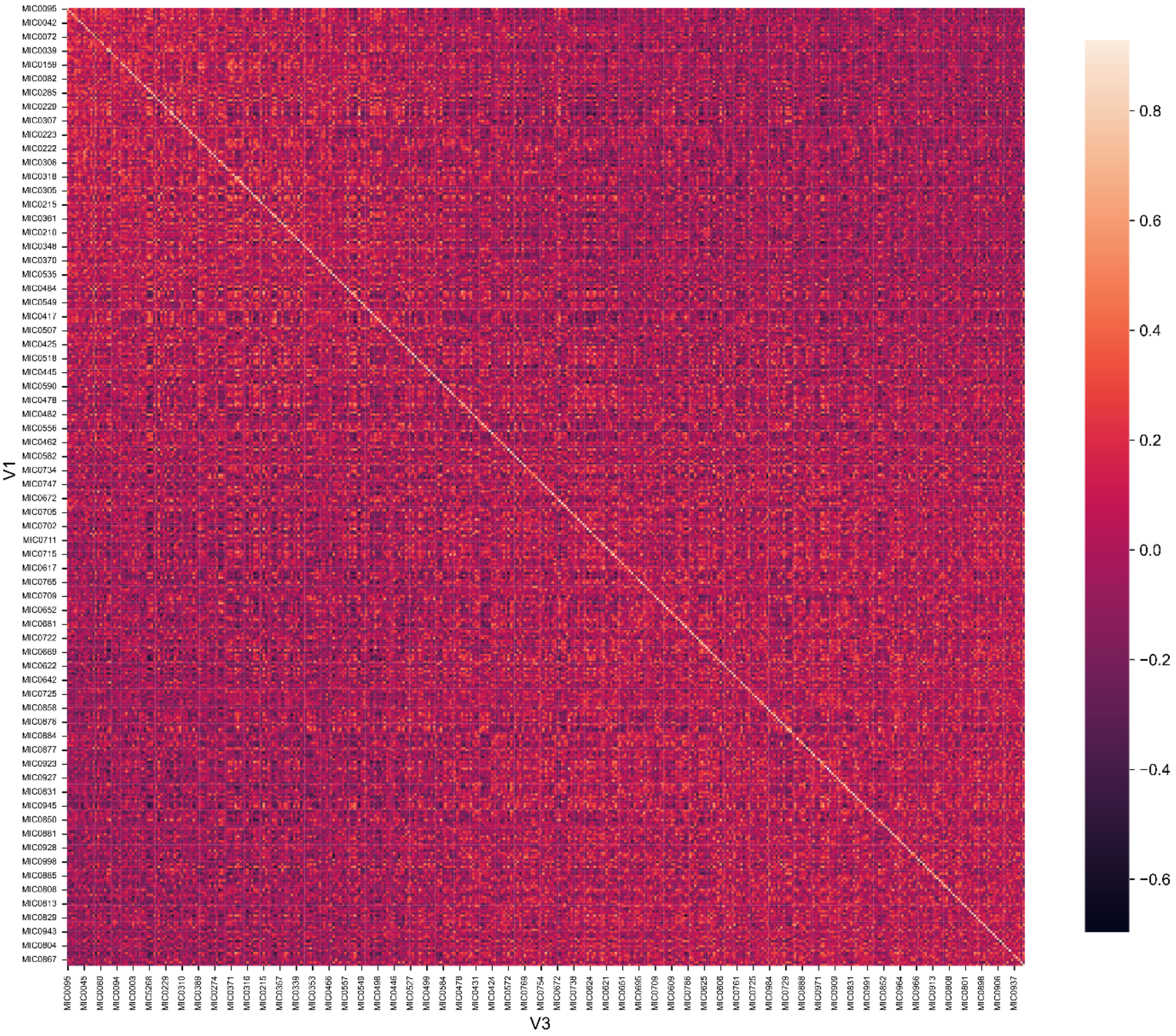

Supplementary Figure 1. Stability of quantitative measurements between V1 and V3 visits across Milieu Intérieur donors. Spearman correlation matrix between unpaired and paired Milieu Intérieur donors, at V1 and V3 visits. The colour gradient indicates the Spearman correlation coefficient. (*n*=405)

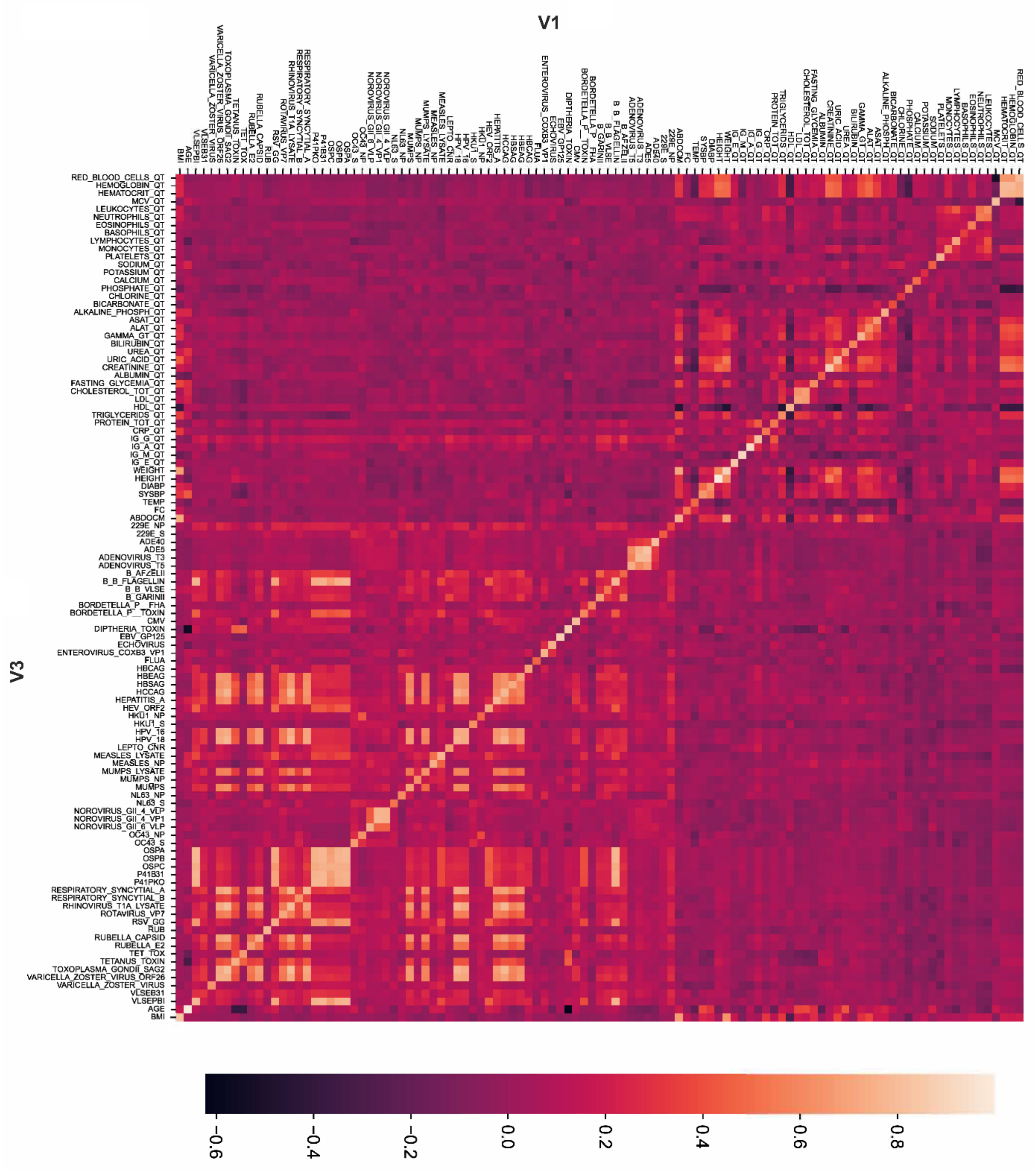

Supplementary Figure 2. Stability of quantitative measurements between V1 and V3 visits across laboratory and serological variables. Spearman correlation matrix between unpaired and paired laboratory and serological measurements, at V1 and V3 visits. The colour gradient indicates the Spearman correlation coefficient (n = 405).

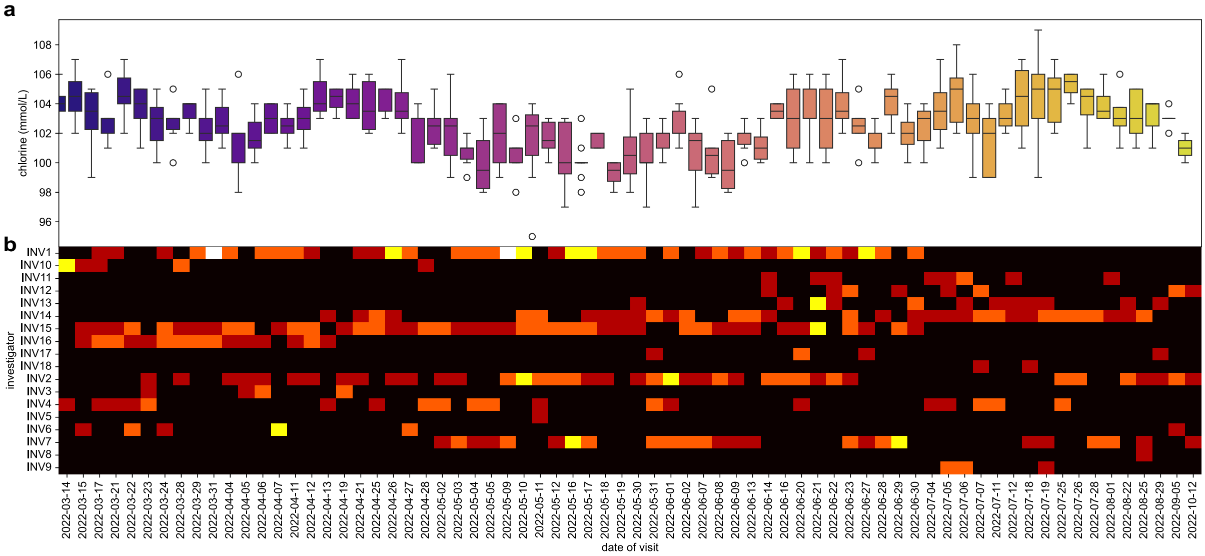

Supplementary Figure 3. Batch effects on chlorine levels. (**a**) Box plot (Median and SDs dhown) of chlorine measurements as a function of the dates of sampling in the V3 visit. (**b**) Heatmap indicating investigators per date of sampling. The colour gradient indicates the number of subjects examined per investigator, at the given date ($n = 405$).

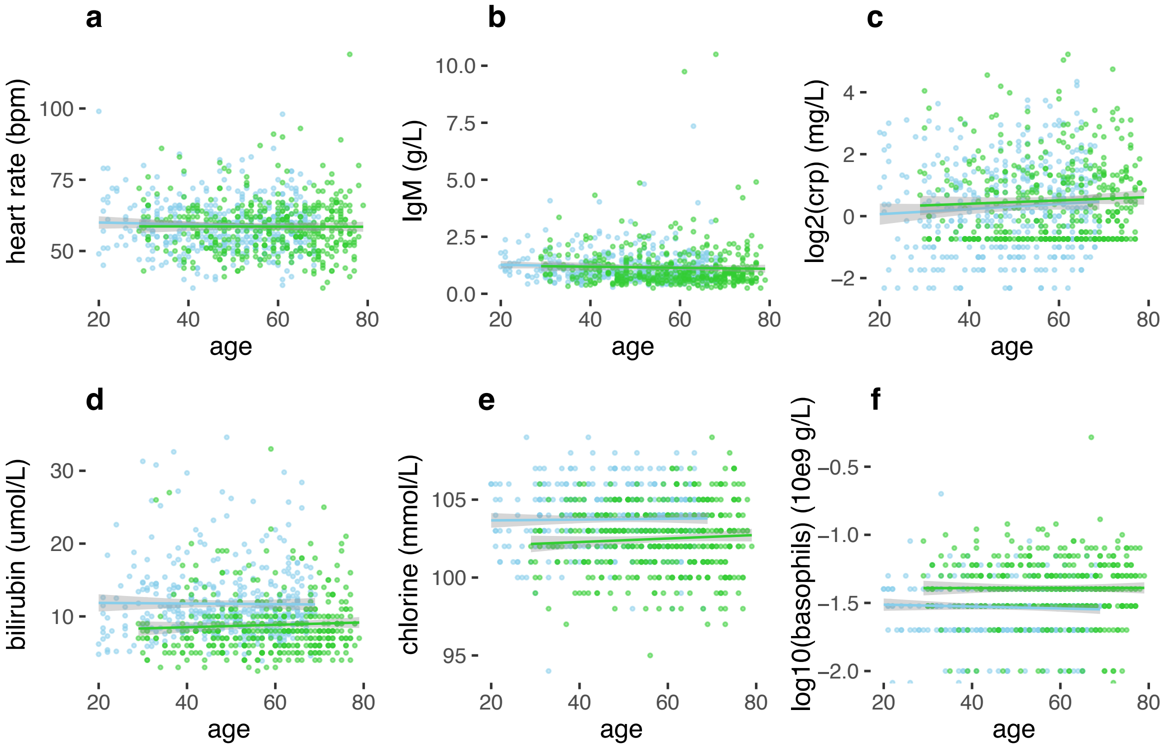

Supplementary Figure 4. Age-independent changes in health biomarkers in the Milieu Intérieur cohort. Scatter plots of (**a**) heart rate, (**b**) immunoglobulin levels, (**c**) $\log_2$-transformed C-reactive protein (CRP) levels, (**d**) bilirubin levels, (**e**) chlorine levels, and (**f**) $\log_{10}$-transformed basophil counts in V1 (blue) and V3 (green) visits, as a function of age. Only variables not associated with age are shown. The solid straight lines indicate the linear regression line. Gray shaded areas indicate the 95% confidence intervals. (n=405)

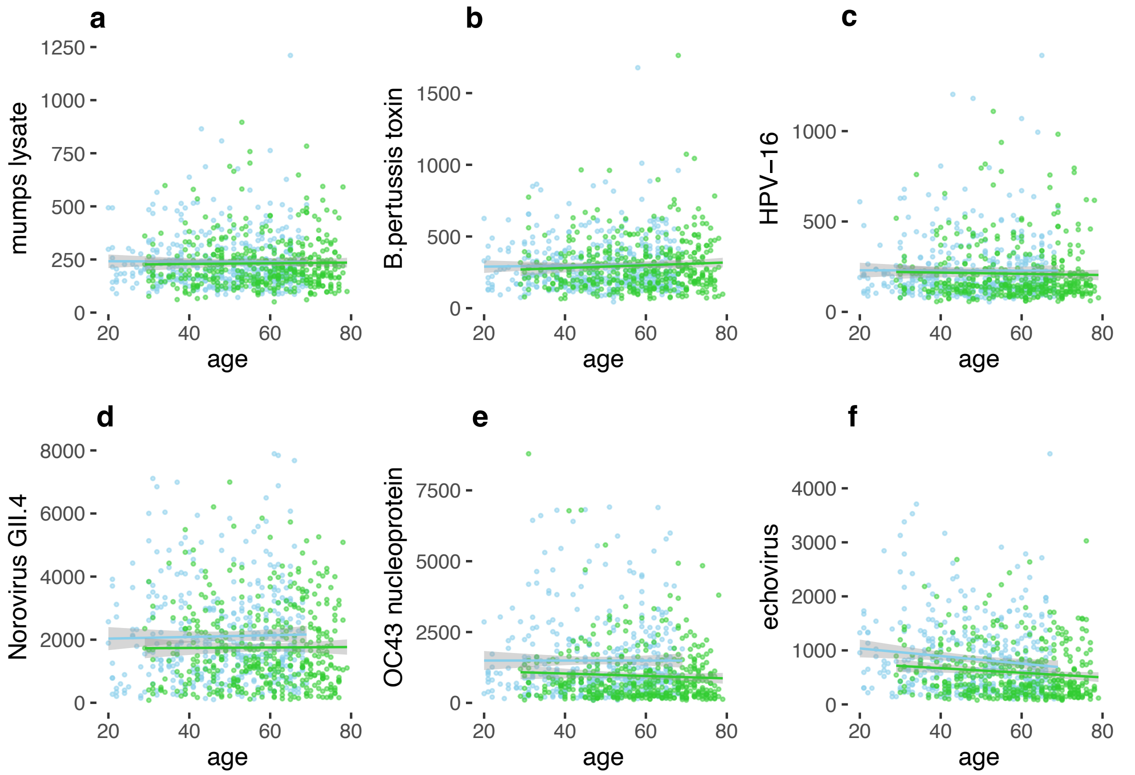

Supplementary Figure 5. Age-independent changes in antibody titers in the Milieu Intérieur follow-up cohort. Scatter plots of Luminex-based MFIs measuring IgGs against (**a**) mumps lysate, (**b**) *Bordetella pertussis* toxin, (**c**) human papillomavirus (HPV) 16, (**d**) norovirus, (**e**) OC43 coronavirus, and (**f**) echovirus in V1 (blue) and V3 (green) visits, as a function of age. Only variables not associated with age are shown. The solid straight line indicates the linear regression line. Gray shared areas indicate the 95% confidence intervals. (*n*=405)
