# Supplementary material for: An integrative approach for studying immunological variation in an aging population – The Milieu Intérieur follow-up study
Source: Nat Commun. 2026 May 22;17:6078. doi: 10.1038/s41467-026-72910-x (PMC13354573; doi:10.1038/s41467-026-72910-x)
Supplement: Supplementary file 2 — Reporting Summary [file 41467_2026_72910_MOESM2_ESM.pdf]

nature portfolio

Corresponding author(s): Darragh Duffy

Last updated by author(s): Mar 20, 2026

Reporting Summary

Nature Portfolio wishes to improve the reproducibility of the work that we publish. This form provides structure for consistency and transparency in reporting. For further information on Nature Portfolio policies, see our Editorial Policies and the Editorial Policy Checklist.

Statistics

For all statistical analyses, confirm that the following items are present in the figure legend, table legend, main text, or Methods section.

n/a	Confirmed	
☐	☒	The exact sample size (*n*) for each experimental group/condition, given as a discrete number and unit of measurement
☐	☒	A statement on whether measurements were taken from distinct samples or whether the same sample was measured repeatedly
☐	☒	The statistical test(s) used AND whether they are one- or two-sided<br>*Only common tests should be described solely by name; describe more complex techniques in the Methods section.*
☐	☒	A description of all covariates tested
☐	☒	A description of any assumptions or corrections, such as tests of normality and adjustment for multiple comparisons
☐	☒	A full description of the statistical parameters including central tendency (e.g. means) or other basic estimates (e.g. regression coefficient) AND variation (e.g. standard deviation) or associated estimates of uncertainty (e.g. confidence intervals)
☐	☒	For null hypothesis testing, the test statistic (e.g. *F*, *t*, *r*) with confidence intervals, effect sizes, degrees of freedom and *P* value noted<br>*Give P values as exact values whenever suitable.*
☒	☐	For Bayesian analysis, information on the choice of priors and Markov chain Monte Carlo settings
☒	☐	For hierarchical and complex designs, identification of the appropriate level for tests and full reporting of outcomes
☒	☐	Estimates of effect sizes (e.g. Cohen's *d*, Pearson's *r*), indicating how they were calculated

Our web collection on statistics for biologists contains articles on many of the points above.

Software and code

Policy information about availability of computer code

Data collection	Statistical tests and multiple regression analyses were conducted in Python 3.10.4, with packages scipy 1.12.0, statsmodel 0.14.0. R software 4.3.0 was also used for Generalized Linear Models (GLM) and Generalized Linear Mixed Models (GLMM) with R packages lmerTest30 3.1-3 and lme431 1.1-35.1. To assess non-linear effects of age, spline regressions and Likelihood-ratio tests were performed with R packages splines 4.3.0 and lmtest 0.9-40. Data visualization was performed using Python package Matplotlib 3.8.0 and Seaborn 0.13.2, or R package ggplot2 3.4.3 and Patchwork 1.2.0.
Data analysis	Statistical tests and multiple regression analyses were conducted in Python 3.10.4, with packages scipy 1.12.0, statsmodel 0.14.0. R software 4.3.0 was also used for Generalized Linear Models (GLM) and Generalized Linear Mixed Models (GLMM) with R packages lmerTest30 3.1-3 and lme431 1.1-35.1. To assess non-linear effects of age, spline regressions and Likelihood-ratio tests were performed with R packages splines 4.3.0 and lmtest 0.9-40. Data visualization was performed using Python package Matplotlib 3.8.0 and Seaborn 0.13.2, or R package ggplot2 3.4.3 and Patchwork 1.2.0.

For manuscripts utilizing custom algorithms or software that are central to the research but not yet described in published literature, software must be made available to editors and reviewers. We strongly encourage code deposition in a community repository (e.g. GitHub). See the Nature Portfolio guidelines for submitting code & software for further information.

Data

Policy information about availability of data

All manuscripts must include a data availability statement. This statement should provide the following information, where applicable:

- Accession codes, unique identifiers, or web links for publicly available datasets
- A description of any restrictions on data availability
- For clinical datasets or third party data, please ensure that the statement adheres to our policy

New CRF, clinical laboratory measures, and serological values are available from the Institut Pasteur Owey data repositories (link) after approval by the Milieu Interieur DAC (requests received at milieuinterieurdac@pasteur.fr). This is a legal requirement for human research studies in France. Previously published data sets including flow cytometry, nanostring gene expression (https://dataset.owey.io/doi/10.48802/owey.vtg6-a567?version=1.0), and Virscan (https://dataset.owey.io/doi/10.48802/owey.84rn-jg72?version=1.1) are available from the same repository at the indicated links.

Research involving human participants, their data, or biological material

Policy information about studies with human participants or human data. See also policy information about sex, gender (identity/presentation), and sexual orientation and race, ethnicity and racism.

Reporting on sex and gender	The MI cohort was stratified for sex and gender. Biological Sex was defined based on presence of X/Y chromosomes as determined using genetic analyses, and gender was defined based on self reporting questionnaire.
Reporting on race, ethnicity, or other socially relevant groupings	As self-reported Metropolitan French origin for three generations was an inclusion criterion of the original study, the majority of the donors are of Western European ancestry, which was confirmed through genetic analyses15.
Population characteristics	*Describe the covariate-relevant population characteristics of the human research participants (e.g. age, genotypic information, past and current diagnosis and treatment categories). If you filled out the behavioural & social sciences study design questions and have nothing to add here, write "See above."*
Recruitment	As this study was designed to be a 10-year follow-up of the original Milieu Intérieur "V1" cohort (NCT01699893 and NCT03905993), the major inclusion criterion was previous inclusion in the V1 study. In addition, donors were required to give written informed consent, and be affiliated to the French social security regimen. Non-inclusion criteria for these pre-screened individuals were restricted to ongoing pregnancy, inability to provide informed consent, or specific reasons requiring legal protective measures. No donors were excluded for any of these non-inclusion criteria. In total, 415 subjects from the previous Milieu Intérieur cohort were included.
Ethics oversight	The 10-year follow-up Milieu Intérieur study, referred to as MI visit 3 ("V3"), was approved by the Comité de Protection des Personnes — Nord Ouest III (Committee for the protection of persons) on 27th January 2022, and by the French Agence nationale de sécurité du médicament (ANSM) on 30th November 2011. The study was sponsored by the Institut Pasteur (ID-RCB Number: 2021-A02621-40)

Note that full information on the approval of the study protocol must also be provided in the manuscript.

Field-specific reporting

Please select the one below that is the best fit for your research. If you are not sure, read the appropriate sections before making your selection.

☒ Life sciences ☐ Behavioural & social sciences ☐ Ecological, evolutionary & environmental sciences

For a reference copy of the document with all sections, see nature.com/documents/nr-reporting-summary-flat.pdf

Life sciences study design

All studies must disclose on these points even when the disclosure is negative.

Sample size	The original MI V1 study was powered to detect medium effect genetic associations. For the MI V3 study presented here we recruited the maximum number of donors that was possible.
Data exclusions	Two donors presented with poor venous capital and one showed evidence for hemolysis, which is known to impact the quality of laboratory measurements. These donors were excluded from biological analyses. One additional donor was excluded because of ongoing breast-feeding. In total, 411 out of 415 donors were kept for biological analysis, with 415 used for questionnaire-based analysis. For all V1 and V3 biological and serological quantitative measurements, only three data points were missing and were imputed with the K-Nearest-Neighbors (K = 5) imputer (scikit-learn package v1.4.1)26. Eleven outlier values, defined as physiologically unlikely data points out of the interval bounded by the first quartile – 4.5 × interquartile range (IQR) and the third quartile + 4.5 × IQR, were replaced by the median of the distribution.<br><br>We found six donors with unexpectedly low correlations between their longitudinal laboratory measures (Spearman's r < 0.30 at FDR = 5%; Fig. 2a and Supplementary Fig. 1), suggesting these V1 and V3 biological data were potentially not collected from the same donor. Although genetic data will be needed to confirm these observations, conservatively we removed the six donors from subsequent analyses
Replication	An equivalent 10 year longitudinal study of healthy donors does not exist to our knowledge to permit replication of our results.

Randomization	Donors were recruited at random in terms of sequence
Blinding	Investigators were not blinded to study groups as there was no experimental intervention

Reporting for specific materials, systems and methods

We require information from authors about some types of materials, experimental systems and methods used in many studies. Here, indicate whether each material, system or method listed is relevant to your study. If you are not sure if a list item applies to your research, read the appropriate section before selecting a response.

Materials & experimental systems

n/a	Involved in the study
☐	☒ Antibodies
☒	☐ Eukaryotic cell lines
☒	☐ Palaeontology and archaeology
☒	☐ Animals and other organisms
☐	☒ Clinical data
☒	☐ Dual use research of concern
☒	☐ Plants

Methods

n/a	Involved in the study
☒	☐ ChIP-seq
☒	☐ Flow cytometry
☒	☐ MRI-based neuroimaging

Antibodies

Antibodies used

Antigen name Short name Optimal coupling concentration (ug/ml) Coupling Buffer Antigen form Expression system Supplier Catalog number
Bordetella pertrussis toxin Bordetella p. toxin 10 PBS Purified toxin Bordetella pertussis The Native Antigen PT-TNL-50
Bordetella pertrussis filamentous haemagglutinin Bordetella p. FHA 5 PBS Purified toxin Bordetella pertussis The Native Antigen BP-FHA-50
Diphtheria toxin Diphtheria toxin 20 PBS Purified toxin Corynebacterium diphtheriae The Native Antigen DIP-TNL-100
Tetanus Toxin Tetanus Toxin (NA) 1 MES Recombinant carboxyl fragment of tetanus toxin heavy chain E.Coli The Native Antigen REC31801-100
Tetanus Toxin Tetanus toxin 14 PBS Purified toxin - NIBSC 02/232
Measles virus lysate Measles lysate 5 PBS Whole virus lysate - The Native Antigen NAT41576-100
Measles virus nucleoprotein Measles nucleoprotein 1 PBS Recombinant protein HEK293 Cells The Native Antigen REC31796-100
Mumps lysate Mumps lysate 5 MES Whole virus lysate - The Native Antigen NAT41577-100
Mumps virus nucleoprotein Mumps nucleoprotein (PS) 50 MES Recombinant protein E.Coli ProSpec-Tany TechnoGene MMP-001
Mumps virus nucleoprotein Mumps nucleoprotein 20 PBS Recombinant protein HEK293 Cells The Native Antigen REC31810-100
Rubella virus-like particles Rubella VLP 4 PBS Virus-like particles HEK293 Cells The Native Antigen REC31651-100
Rubella virus Capsid Rubella Capsid 2,5 MES Recombinant protein E.Coli ProSpec-Tany TechnoGene RUB-293
Rubella virus E2 Rubella E2 1 MES Recombinant protein E.Coli ProSpec-Tany TechnoGene RUB-292
Hepatitis B Virus E antigen Hepatitis B E antigen 1 MES Recombinant protein E.Coli The Native Antigen REC31677-100
Hepatitis B Virus Core antigen Hepatitis B core antigen 5 PBS Recombinant protein E.Coli The Native Antigen REC31689-100
Hepatitis B Virus Surface antigen Hepatitis B surface antigen 1 PBS Recombinant protein Pichia Pastoris ProSpec-Tany TechnoGene HBS-872
Human Papillomavirus serotype 16 HPV 16 40 MES Recombinant protein E.Coli ProSpec-Tany TechnoGene HPV-001
Human Papillomavirus serotype 18 HPV 18 2,5 MES Recombinant protein E.Coli ProSpec-Tany TechnoGene HPV-002
Varicella-Zoster Virus, Heterodimer gE/Gi Varicella-Zoster Virus 5 PBS Recombinant protein HEK293 Cells The Native Antigen REC31907-100
Adenovirus T3 Adenovirus T3 lysate 20 MES Whole virus lysate - The Native Antigen AD004-100
Adenovirus T5 Adenovirus T5 lysate 20 MES Whole virus lysate - The Native Antigen AD005-100
Adenovirus Type 5 Hexon Protein Adenovirus T5 20 PBS Purified hexon protein - The Native Antigen AH01-100
Adenovirus Type 40 Hexon Protein Adenovirus T40 20 PBS Purified hexon protein - The Native Antigen NAT41552-100
Cytomegalovirus strain AD-169 Cytomegalovirus 5 MES Purified virus particles - The Native Antigen CMV-HP-100
Epstein-Barr Virus protein BALF4 Epstein-Barr virus 5 MES Recombinant protein - The Native Antigen REC31601-100
Echovirus Echovirus 10 PBS Recombinant protein E.Coli The Native Antigen REC31776-100
Enterovirus CoxB3 VP1 Enterovirus CoxB3 VP1 10 PBS Recombinant protein E.Coli The Native Antigen REC31738-10
Hepatitis A Virus Hepatitis A 200 MES Recombinant protein FRhk-4 Cells RayBiotech 227-10025
Hepatitis C Core antigen Hepatitis C Core antigen 1 MES Recombinant protein E.Coli The Native Antigen REC31693-100
Hepatitis E Virus ORF2 Hepatitis E ORF2 10 PBS Recombinant protein HEK293 Cells The Native Antigen REC31653-100
Norovirus GII.4 VP1 Norovirus GII.4 VP1 20 MES Recombinant protein HEK293 Cells The Native Antigen REC32015-100
Norovirus GII.6 VLP Norovirus GII.6 2,5 MES Virus-Like Particles HEK293 Cells The Native Antigen REC31985-100
Respiratory Syncytial virus A lysate Respiratory Syncytial virus A 1 MES Whole virus lysate - The Native Antigen NAT41624-100
Respiratory Syncytial virus B Respiratory Syncytial virus B 2,5 MES Whole virus lysate - The Native Antigen NAT41625-100 lysate
Respiratory Syncytial virus glycoprotein G Respiratory Syncytial virus gG 2,5 PBS Recombinant protein HEK293 Cells The Native Antigen RSV-GPB-50

Rhinovirus type 1A lysate Rhinovirus T1A 1 MES Whole virus lysate - The Native Antigen NAT41626-100

Rotavirus VP7 Rotavirus VP7 5 MES Recombinant protein HEK293 Cells The Native Antigen REC31910-100

Human coronavirus OC43 nucleoprotein OC43 nucleoprotein 10 PBS Recombinant protein E.Coli The Native Antigen REC31857-100

Human coronavirus OC43 Spike protein OC43 spike 5 PBS Recombinant protein HEK293 Cells The Native Antigen REC31894-100

Human coronavirus HKU1 nucleoprotein HKU1 nucleoprotein 10 PBS Recombinant protein E.Coli The Native Antigen REC31856-100

Human coronavirus HKU1 Spike protein HKU1 spike 5 PBS Recombinant protein HEK293 Cells The Native Antigen REC31897-100

Human coronavirus 229E nucleoprotein 229E nucleoprotein 10 PBS Recombinant protein E.Coli The Native Antigen REC31758-100

Human coronavirus 229E Spike protein 229E spike 10 PBS Recombinant protein HEK293 Cells The Native Antigen REC31895-100

Human coronavirus NL63 nucleoprotein NL63 nucleoprotein 10 PBS Recombinant protein E.Coli The Native Antigen REC31759-100

Human coronavirus NL63 Spike protein NL63 spike 5 PBS Recombinant protein HEK293 Cells The Native Antigen REC31896-100

Influenza A H1N1 Hemagglutinin Influenzavirus A 20 PBS Recombinant protein HEK293 Cells The Native Antigen FLUH1N1-HA-100

SARS-CoV-2 Spike Wuhan SARS-CoV-2 Spike Wuhan 10 PBS Recombinant protein HEK293 Cells Institut Pasteur

SARS-CoV-2 Spike alpha SARS-CoV-2 Spike alpha 10 PBS Recombinant protein HEK293 Cells Institut Pasteur

SARS-CoV-2 Spike beta SARS-CoV-2 Spike beta 10 PBS Recombinant protein HEK293 Cells Institut Pasteur

SARS-CoV-2 Spike delta SARS-CoV-2 Spike delta 10 PBS Recombinant protein HEK293 Cells Institut Pasteur

SARS-CoV-2 Receptor Binding Domain Wuhan SARS-CoV-2 RBD Wuhan 10 PBS Recombinant protein HEK293 Cells Institut Pasteur

SARS-CoV-2 Receptor Binding Domain alpha SARS-CoV-2 RBD alpha 10 PBS Recombinant protein HEK293 Cells Institut Pasteur

SARS-CoV-2 Receptor Binding Domain beta SARS-CoV-2 RBD beta 10 PBS Recombinant protein HEK293 Cells Institut Pasteur

SARS-CoV-2 Receptor Binding Domain delta SARS-CoV-2 RBD delta 10 PBS Recombinant protein HEK293 Cells Institut Pasteur

SARS-CoV-2 nuceloprotein SARS-CoV-2 NP 10 PBS Recombinant protein HEK293 Cells Institut Pasteur

SARS-CoV-2 Spike subunit 2 SARS-CoV-2 S2 10 PBS Recombinant protein HEK293 Cells The Native Antigen REC31807

SARS-CoV-2 Membrane Envelope SARS-CoV-2 ME 10 PBS Recombinant protein HEK293 Cells The Native Antigen REC31829

Validation

Antigen name Short name Optimal coupling concentration (ug/ml) Coupling Buffer Antigen form Expression system Supplier Catalog number

Bordetella pertrussis toxin Bordetella p. toxin 10 PBS Purified toxin Bordetella pertussis The Native Antigen PT-TNL-50

Bordetella pertrussis filamentous haemagglutinin Bordetella p. FHA 5 PBS Purified toxin Bordetella pertussis The Native Antigen BP-FHA-50

Diphtheria toxin Diphtheria toxin 20 PBS Purified toxin Corynebacterium diphtheriae The Native Antigen DIP-TNL-100

Tetanus Toxin Tetanus Toxin (NA) 1 MES Recombinant carboxyl fragment of tetanus toxin heavy chain E.Coli The Native Antigen REC31801-100

Tetanus Toxin Tetanus toxin 14 PBS Purified toxin - NIBSC 02/232

Measles virus lysate Measles lysate 5 PBS Whole virus lysate - The Native Antigen NAT41576-100

Measles virus nucleoprotein Measles nucleoprotein 1 PBS Recombinant protein HEK293 Cells The Native Antigen REC31796-100

Mumps lysate Mumps lysate 5 MES Whole virus lysate - The Native Antigen NAT41577-100

Mumps virus nucleoprotein Mumps nucleoprotein (PS) 50 MES Recombinant protein E.Coli ProSpec-Tany TechnoGene MMP-001

Mumps virus nucleoprotein Mumps nucleoprotein 20 PBS Recombinant protein HEK293 Cells The Native Antigen REC31810-100

Rubella virus-like particles Rubella VLP 4 PBS Virus-like particles HEK293 Cells The Native Antigen REC31651-100

Rubella virus Capsid Rubella Capsid 2,5 MES Recombinant protein E.Coli ProSpec-Tany TechnoGene RUB-293

Rubella virus E2 Rubella E2 1 MES Recombinant protein E.Coli ProSpec-Tany TechnoGene RUB-292

Hepatitis B Virus E antigen Hepatitis B E antigen 1 MES Recombinant protein E.Coli The Native Antigen REC31677-100

Hepatitis B Virus Core antigen Hepatitis B core antigen 5 PBS Recombinant protein E.Coli The Native Antigen REC31689-100

Hepatitis B Virus Surface antigen Hepatitis B surface antigen 1 PBS Recombinant protein Pichia Pastoris ProSpec-Tany TechnoGene HBS-872

Human Papillomavirus serotype 16 HPV 16 40 MES Recombinant protein E.Coli ProSpec-Tany TechnoGene HPV-001

Human Papillomavirus serotype 18 HPV 18 2,5 MES Recombinant protein E.Coli ProSpec-Tany TechnoGene HPV-002

Varicella-Zoster Virus, Heterodimer gE/Gi Varicella-Zoster Virus 5 PBS Recombinant protein HEK293 Cells The Native Antigen REC31907-100

Adenovirus T3 Adenovirus T3 lysate 20 MES Whole virus lysate - The Native Antigen AD004-100

Adenovirus T5 Adenovirus T5 lysate 20 MES Whole virus lysate - The Native Antigen AD005-100

Adenovirus Type 5 Hexon Protein Adenovirus T5 20 PBS Purified hexon protein - The Native Antigen AH01-100

Adenovirus Type 40 Hexon Protein Adenovirus T40 20 PBS Purified hexon protein - The Native Antigen NAT41552-100

Cytomegalovirus strain AD-169 Cytomegalovirus 5 MES Purified virus particles - The Native Antigen CMV-HP-100

Epstein-Barr Virus protein BALF4 Epstein-Barr virus 5 MES Recombinant protein - The Native Antigen REC31601-100

Echovirus Echovirus 10 PBS Recombinant protein E.Coli The Native Antigen REC31776-100

Enterovirus CoxB3 VP1 Enterovirus CoxB3 VP1 10 PBS Recombinant protein E.Coli The Native Antigen REC31738-10

Hepatitis A Virus Hepatitis A 200 MES Recombinant protein FRhk-4 Cells RayBiotech 227-10025

Hepatitis C Core antigen Hepatitis C Core antigen 1 MES Recombinant protein E.Coli The Native Antigen REC31693-100

Hepatitis E Virus ORF2 Hepatitis E ORF2 10 PBS Recombinant protein HEK293 Cells The Native Antigen REC31653-100

Norovirus GII.4 VP1 Norovirus GII.4 VP1 20 MES Recombinant protein HEK293 Cells The Native Antigen REC32015-100

Norovirus GII.6 VLP Norovirus GII.6 2,5 MES Virus-Like Particles HEK293 Cells The Native Antigen REC31985-100

Respiratory Syncytial virus A lysate Respiratory Syncytial virus A 1 MES Whole virus lysate - The Native Antigen NAT41624-100

Respiratory Syncytial virus B Respiratory Syncytial virus B 2,5 MES Whole virus lysate - The Native Antigen NAT41625-100
lysate

Respiratory Syncytial virus glycoprotein G Respiratory Syncytial virus gG 2,5 PBS Recombinant protein HEK293 Cells The Native Antigen RSV-GPB-50

Rhinovirus type 1A lysate Rhinovirus T1A 1 MES Whole virus lysate - The Native Antigen NAT41626-100

Rotavirus VP7 Rotavirus VP7 5 MES Recombinant protein HEK293 Cells The Native Antigen REC31910-100

Human coronavirus OC43 nucleoprotein OC43 nucleoprotein 10 PBS Recombinant protein E.Coli The Native Antigen REC31857-100

Human coronavirus OC43 Spike protein OC43 spike 5 PBS Recombinant protein HEK293 Cells The Native Antigen REC31894-100
Human coronavirus HKU1 nucleoprotein HKU1 nucleoprotein 10 PBS Recombinant protein E.Coli The Native Antigen REC31856-100
Human coronavirus HKU1 Spike protein HKU1 spike 5 PBS Recombinant protein HEK293 Cells The Native Antigen REC31897-100
Human coronavirus 229E nucleoprotein 229E nucleoprotein 10 PBS Recombinant protein E.Coli The Native Antigen REC31758-100
Human coronavirus 229E Spike protein 229E spike 10 PBS Recombinant protein HEK293 Cells The Native Antigen REC31895-100
Human coronavirus NL63 nucleoprotein NL63 nucleoprotein 10 PBS Recombinant protein E.Coli The Native Antigen REC31759-100
Human coronavirus NL63 Spike protein NL63 spike 5 PBS Recombinant protein HEK293 Cells The Native Antigen REC31896-100
Influenza A H1N1 Hemagglutinin Influenzavirus A 20 PBS Recombinant protein HEK293 Cells The Native Antigen FLUH1N1-HA-100
SARS-CoV-2 Spike Wuhan SARS-CoV-2 Spike Wuhan 10 PBS Recombinant protein HEK293 Cells Institut Pasteur
SARS-CoV-2 Spike alpha SARS-CoV-2 Spike alpha 10 PBS Recombinant protein HEK293 Cells Institut Pasteur
SARS-CoV-2 Spike beta SARS-CoV-2 Spike beta 10 PBS Recombinant protein HEK293 Cells Institut Pasteur
SARS-CoV-2 Spike delta SARS-CoV-2 Spike delta 10 PBS Recombinant protein HEK293 Cells Institut Pasteur
SARS-CoV-2 Receptor Binding Domain Wuhan SARS-CoV-2 RBD Wuhan 10 PBS Recombinant protein HEK293 Cells Institut Pasteur
SARS-CoV-2 Receptor Binding Domain alpha SARS-CoV-2 RBD alpha 10 PBS Recombinant protein HEK293 Cells Institut Pasteur
SARS-CoV-2 Receptor Binding Domain beta SARS-CoV-2 RBD beta 10 PBS Recombinant protein HEK293 Cells Institut Pasteur
SARS-CoV-2 Receptor Binding Domain delta SARS-CoV-2 RBD delta 10 PBS Recombinant protein HEK293 Cells Institut Pasteur
SARS-CoV-2 nuceloprotein SARS-CoV-2 NP 10 PBS Recombinant protein HEK293 Cells Institut Pasteur
SARS-CoV-2 Spike subunit 2 SARS-CoV-2 S2 10 PBS Recombinant protein HEK293 Cells The Native Antigen REC31807
SARS-CoV-2 Membrane Envelope SARS-CoV-2 ME 10 PBS Recombinant protein HEK293 Cells The Native Antigen REC31829

Clinical data

Policy information about clinical studies

All manuscripts should comply with the ICMJE guidelines for publication of clinical research and a completed CONSORT checklist must be included with all submissions.

Clinical trial registration: NCT05381857

Study protocol: NCT05381857

Data collection: Donors were interviewed and sampled during a single visit at Biotrial (Rennes, France) between the period of 14th March 2022 and 12th October 2022.

Outcomes: Outcomes were related to assessment of variable immune responses and associations with genetic and environmental factors

Plants

Seed stocks: *Report on the source of all seed stocks or other plant material used. If applicable, state the seed stock centre and catalogue number. If plant specimens were collected from the field, describe the collection location, date and sampling procedures.*

Novel plant genotypes: *Describe the methods by which all novel plant genotypes were produced. This includes those generated by transgenic approaches, gene editing, chemical/radiation-based mutagenesis and hybridization. For transgenic lines, describe the transformation method, the number of independent lines analyzed and the generation upon which experiments were performed. For gene-edited lines, describe the editor used, the endogenous sequence targeted for editing, the targeting guide RNA sequence (if applicable) and how the editor was applied.*

Authentication: *Describe any authentication procedures for each seed stock used or novel genotype generated. Describe any experiments used to assess the effect of a mutation and, where applicable, how potential secondary effects (e.g. second site T-DNA insertions, mosiacism, off-target gene editing) were examined.*
