# Supplementary material for: An integrative approach for studying immunological variation in an aging population – The Milieu Intérieur follow-up study
Source: Nat Commun. 2026 May 22;17:6078. doi: 10.1038/s41467-026-72910-x (PMC13354573; doi:10.1038/s41467-026-72910-x)
Supplement: Supplementary file 3 — Transparent Peer Review file [file 41467_2026_72910_MOESM3_ESM.pdf]

nature portfolio

Peer Review File

The Milieu Intérieur follow-up study – an integrative approach for studying immunological variation in an aging population

Corresponding Author: Dr Darragh DUFFY

This file contains all reviewer reports in order by version, followed by all author rebuttals in order by version.

Version 0:

Reviewer comments:

Reviewer #1

(Remarks to the Author)
The Milieu Intérieur follow-up study – an integrative approach for studying
immunological variation in an aging population
Anthony Jaquaniello et al.

The study was designed to address an important question of changes in the baseline state of the immune system as it evolves over a 10 year period, which encompassed the pandemic. Authors analyzed data from over 400 participants, and collected over a hundred measures, that were expected to reveal the dynamics of aging of the immune system. Given the wealth of information collected, it is unexpected and unfortunate that it provided little to improve our understanding of immune system evolution over time. Indeed, the most striking observation made was the correlation between the PhenoAge at the time of recruitment (V1) was strongly correlated with disease development at V3 (10 years later). However, PhenoAge itself strongly correlates with actual age, so the practical value of this observation can be put into question. Another interesting piece of information related to the changes in serological readouts (antibody titers) against a large panel of common pathogens (coronavirus, adenovisrus etc) and vaccine antigens (measles, mumps etc) over time. Unfortunately, this piece of data was relegated to supplementary figures.

Overall, the reader is compelled to appreciate the tremendous amount of effort and resources that were spent on the recruitment of such a large cohort, 10 year follow up, collection and analysis of such a large number of samples, and the tremendous wealth of information hidden in this dataset. Unfortunately, the authors fall short of the mark when it comes to mining this unique body of data for truly clinically pertinent information, or for novel findings that would improve our understanding of the mechanisms that drive the evolution of human body, specifically the immune system, over time. The results are presented in a rather descriptive manner, with no clear focus on the take-home message. Presented in this way, the data lacks novelty and impact that would be expected from a Nature Communications paper. It would be great if the authors could find a way re-frame their approach to dealing with the large and diverse dataset, and to re-focus the paper on a few truly important and novel findings. Substantial visual improvements to figures would be also desirable. Unfortunately, in its current form the manuscript is not suitable for publication in Nature Communications.

Reviewer #2

(Remarks to the Author)
The authors present a follow-up on the important Milieu Intérieur cohort, which included 1,000 healthy adults and from which much biological data pertaining to health and immunity has been derived. This 10-year follow-up comprises 415 of the original 1,000 participants, who provided questionnaire data, blood samples and nasal swabs. A wide panel of pathogen-specific antibodies were screened in serum, along with other pertinent measures such as routine clinical data and physical assessments. Comparison of demographic and assessment data between time points suggested that health did decline in these participants, although it is not clear to me whether selection bias truly wasn't apparent (see below). This is critical given that the method of recruitment is very susceptible to this risk. The authors go on to report significant trajectories of routine biological measures, pathogen exposure, and disease incidence. They also report changes in serum PhenoAge over time, finding differences between time points when considering disease status, which I am assuming represents any co-morbid condition. These comparisons weren't exactly clear either, as detailed below. While the manuscript is very well

written, and the research in exceptional in its magnitude, the findings mostly lack novelty, compounded by concerns regarding the selection bias. I also am not sure the title, which implies that “immunological variation” was studied here, really fits the data presented. My additional comments are below. Thank you for providing me the opportunity to review this work, I hope it is helpful.

Comments

Line 211 – I am having trouble understanding the comparisons performed to discount potential selection bias for the cohort of 415. Figure 1B appears to be comparing the age of all participants at V1 vs. the participants at V2, showing age to be significantly higher, which is expected. What was the difference at V1 for those that ended up in V3 and those that did not? The comparison seems to be described this way for the SES factors, and does not appear to be different. Nonetheless, having a detailed summary table in the supplementary comparing demographic, lifestyle, SES and assessment measures at V1 for those that did and did not participate in V3 would be very helpful to truly be confident regarding the degree of selection bias (or lack thereof) for this follow-up study.

Line 346 – The analysis of PhenoAge over time is very exciting, but the manner in which it is described in the text and reported in Figure 5 is unclear to me. It appears to indicate that higher PhenoAge at V1 is related to the incidence of disease at V3. Is that correct? How was that modelled? I am surprised that a change score model was not implemented wherein a participant’s PhenoAge at V3 was regressed against their PhenoAge at V1 along with additional fixed effects representing other factors measured (e.g. SES, diseases, sex, etc.). Even serostatus for various pathogens could be included in such an analysis allowing for conclusions to be drawn regarding the role of infectious load in altering biological age trajectories.

Reviewer #3

(Remarks to the Author)
In the manuscript "The Milieu Intérieur follow-up study – an integrative approach for studying immunological variation in an aging population," Jaquaniello et al. present results from a 10-year follow-up clinical study of a healthy cohort from the French population, aiming to identify biomarkers associated with the aging process. The study is well-designed and performed.

Below are some minor remarks:

• Although the authors present an approach to studying how the immune system changes with age, the manuscript would benefit from a more detailed discussion of their results and their implications for aging. For example, in the last sub-section of the Results section, they mention that the "PhenoAge score was strongly correlated with chronological age," but the direction of changes in albumin, creatinine, glucose, and other biomarkers is not clear. It would also be interesting if the authors discuss how the observed changes can be related to health status and what their relation is to disease and aging itself.

• There are statements in the Results section that are not linked to corresponding tables or figures. For example, in line 329, the authors state, "We found that the disease group was significantly older than the other groups (p=0.017)," but they do not cite the table in which these data are presented, making it unclear for the reader to see the exact difference between the groups. This issue occurs throughout the text, so please review and ensure proper citation of tables and figures.

• In lines 239 and 245, the authors present data from correlation analyses with false discovery rate correction of the p-values. This should be mentioned in the "Statistical analyses and data visualization" section.

• The manuscript discusses physiological processes and biomarkers that can be influenced by various factors, including genetic background. Therefore, it is important to include information about the ethnicity of the cohort in the Materials and Methods section. Additionally, if the cohort consists of individuals from different ethnic backgrounds, this should be discussed accordingly.

Reviewer #4

(Remarks to the Author)
I co-reviewed this manuscript with one of the reviewers who provided the listed reports. This is part of the Nature Communications initiative to facilitate training in peer review and to provide appropriate recognition for Early Career Researchers who co-review manuscripts.

Version 1:

Reviewer comments:

Reviewer #2

(Remarks to the Author)
Many thanks to the authors for considering my comments. I believe the new analyses and figures added do a nice job of

confirming that selection bias wasn't a large issue.

Regarding the revisions to the PhenoAge analysis, I am still unclear what was done. In your rebuttal letter you state: "This association was tested using a logistic model in which disease status at V3 was modelled as a function of PhenoAge at V1, adjusting for chronological age at V3". First, it is common practice to report the odds ratio from a logistic regression model, and allows for comparability of estimates across studies. Second, although a logistic regression model was used, lines 277 and 288 report a t-test p-value. Please clarify. Lastly, the statement "We have not reported results from models with additional fixed effects, as none of them were significantly associated with disease status" is somewhat concerning given that one would expect variables such as sex, BMI and SES to be associated with the incidence of disease after ten years. If the authors can confirm that this is indeed the case, it would be important to mention as a limitation of this data, as it suggests the participants involved were likely derived from a low risk population and are not representative of the general population. This seems to be the case from the PhenoAge vs Chronological age scatterplot, since the entire cohort has a starting PhenoAge that is less than zero. I don't believe this is mentioned in the text, but please correct me if I am wrong.

Reviewer #3

(Remarks to the Author)
I would like to thank the authors for their thorough and thoughtful revision of the manuscript. They have successfully addressed all the major and minor points raised in the previous round of reviews. The manuscript is now significantly strengthened, and the narrative is much clearer, more impactful, and better supported by the data presented. The manuscript, in its current form, contributes to our understanding of immune aging and the identification of biomarkers for future health outcomes.

Reviewer #4

(Remarks to the Author)
I co-reviewed this manuscript with one of the reviewers who provided the listed reports. This is part of the Nature Communications initiative to facilitate training in peer review and to provide appropriate recognition for Early Career Researchers who co-review manuscripts.

Reviewer #5

(Remarks to the Author)
This is a well-written, revised manuscript detailing a number of analyses using an incredibly rich dataset from a well-characterized population. This cohort and it's data should provide unique opportunities to explore mechanisms of human aging. The revised manuscript includes several new analyses to bear further investigation.

The revised manuscript has several additions in response to reviewer concerns. Most of the reviewer concerns were addressed, although there are still some unanswered questions.
• Additional findings linking gene expression in response to microbial stimulation in vitro (a surrogate marker for immune functionality) with disease occurrence.
• A more complete V3 donor characterization. Results suggest that there is minimal selection bias.
• Virscan analysis highlighted several antibody responses to CMV proteins. Conclusion is that there is a general loss of Ab response to all antigens in participants who serorevert.
• Clarifications on the PhenoAge analysis. The response to reviewers indicates that the PhenoAge analysis did not include additional fixed effects because they were not associated with disease status. This is not explicitly stated in the section outlining PhenoAge results (lines 266-280).
• Results statements in the Discussion are still not linked to figures/tables.

A new author was added to the revised manuscript. Most of the author change forms lack printed names and those signatures cannot be linked to the authors. Furthermore, several forms have the same signature(s) on them. These documents need to be resubmitted with printed names to ensure that all of the original authors agreed to the authorship change.

Was vaccination history between V1 and V3 collected? This may explain some of the immunologic changes. Also, why was mumps seroconversion so high? This was not discussed in the text and is at least as surprising as the similar B pertussis findings.

Line 204-205 the authors suggest that their data support revaccination campaigns because there are changes in Ab titer over time. This would be a stronger statement if there was a decrease in seroprotection with age rather than simply 'dynamic changes in Ab titer with age'. Defined correlates of protection exist for several of the pathogens tested.

The immune stimulation response being linked to disease development is one of the stronger and more interesting findings. The authors should discuss why some stimulations conditions seem 'better' (correlated with disease development) than others. Also, it is not the response to stimulus that is correlated with disease, it is the change in response to stimulus (V1 versus V3) that is correlated (See Figure 6a) – this needs to be more clearly stated in the Discussion.

Table 3 – define 'at-risk'.

Version 2:

Reviewer comments:

Reviewer #2

(Remarks to the Author)
Thank you for considering my comments. I am satified with the changes made and have no additional feedback.

Reviewer #5

(Remarks to the Author)
The authors have responded to all of the reviewer comments and have clarified the important points.

Open Access This Peer Review File is licensed under a Creative Commons Attribution 4.0 International License, which permits use, sharing, adaptation, distribution and reproduction in any medium or format, as long as you give appropriate credit to the original author(s) and the source, provide a link to the Creative Commons license, and indicate if changes were made.
In cases where reviewers are anonymous, credit should be given to 'Anonymous Referee' and the source.
The images or other third party material in this Peer Review File are included in the article's Creative Commons license, unless indicated otherwise in a credit line to the material. If material is not included in the article's Creative Commons license and your intended use is not permitted by statutory regulation or exceeds the permitted use, you will need to obtain permission directly from the copyright holder.
To view a copy of this license, visit https://creativecommons.org/licenses/by/4.0/

We thank the reviewers for their very helpful comments, below is a detailed, point-by-point response.

Sincerely
Darragh Duffy

REVIEWER COMMENTS

Reviewer #1 (Remarks to the Author):

The Milieu Intérieur follow-up study – an integrative approach for studying immunological variation in an aging population
Anthony Jaquaniello et al.

The study was designed to address an important question of changes in the baseline state of the immune system as it evolves over a 10 year period, which encompassed the pandemic. Authors analyzed data from over 400 participants, and collected over a hundred measures, that were expected to reveal the dynamics of aging of the immune system. Given the wealth of information collected, it is unexpected and unfortunate that it provided little to improve our understanding of immune system evolution over time. Indeed, the most striking observation made was the correlation between the PhenoAge at the time of recruitment (V1) was strongly correlated with disease development at V3 (10 years later). However, PhenoAge itself strongly correlates with actual age, so the practical value of this observation can be put into question. Another interesting piece of information related to the changes in serological readouts (antibody titers) against a large panel of common pathogens (coronavirus, adenovisrus etc) and vaccine antigens (measles, mumps etc) over time. Unfortunately, this piece of data was relegated to supplementary figures.

Overall, the reader is compelled to appreciate the tremendous amount of effort and resources that were spent on the recruitment of such a large cohort, 10 year follow up, collection and analysis of such a large number of samples, and the tremendous wealth of information hidden in this dataset. Unfortunately, the authors fall short of the mark when it comes to mining this unique body of data for truly clinically pertinent information, or for novel findings that would improve our understanding of the mechanisms that drive the evolution of human body, specifically the immune system, over time. The results are presented in a rather descriptive manner, with no clear focus on the take-home message. Presented in this way, the data lacks novelty and impact that would be expected from a Nature Communications paper. It would be great if the authors could find a way re-frame their approach to dealing with the large and diverse dataset, and to re-focus the paper on a few truly important and novel findings. Substantial visual improvements to figures would be also desirable. Unfortunately, in its current form the manuscript is not suitable for publication in Nature Communications.

- **We thank the reviewer for their careful read and thoughtful evaluation of our manuscript, as well as for acknowledging the effort involved in this study.**

We understand the concern that the initial version lacked truly clinically relevant information. <u>To address this, we have now performed extensive new data analysis</u>. We now report the important finding that specific immune gene expression signatures, induced after whole blood stimulation with *Staphylococcus aureus* or *Candida albicans*, are predictive of disease development over a 10-year period. Strikingly, none of these genes showed differential expression between healthy and disease groups in the absence of immune stimulation, nor were significant differences observed in circulating immune cell phenotypes. This finding demonstrates the power of such an in-depth phenotyped longitudinal human cohort and demonstrates the potential of using functional immune responses as predictive biomarkers of disease. <u>These results are now presented in a new Figure 6 and are a major focus of the revised discussion.</u>

- **In addition, we have expanded our previous analysis to include a more detailed characterization of V3 donors versus the original non-participants (as requested by reviewer 2 to address questions related to recruitment bias).**

- **We have also expanded the serological analysis to better document cases of seroconversion and seroreversion over the 10-year period. Notably, we observe unexpected cases of CMV seroreversion, which is surprising given the virus' well-known ability to remain latent. To further strengthen this observation, we leverage a novel Virscan data set from the V1 visit (part of an independent study currently under review; Preprint reference is cited), showing that donors who seroreverted for CMV at V3 had lower antibody levels against a broad array of viral epitopes at V1.**

- **Utilizing the same Virscan dataset, we have also expanded our analysis of the cohort during the Covid-19 pandemic. We have tested the hypothesis that prior immunity to seasonal coronaviruses may impact antibody responses to either infection or vaccination against SARS-CoV-2—an effect previously reported over shorter time periods. Our results suggest that this effect does not persist over longer periods such as 10 years.**

- **Finally, we have substantially modified the manuscript to address the reviewer's concern regarding a lack of clear focus or take-home message. We sincerely thank the reviewer for these comments, which we believe have helped us significantly improve the clarity and impact of the manuscript.**

Reviewer #2 (Remarks to the Author):

The authors present a follow-up on the important Milieu Intérieur cohort, which included 1,000 healthy adults and from which much biological data pertaining to health and immunity has been derived. This 10-year follow-up comprises 415 of the original 1,000 participants, who provided questionnaire data, blood samples and nasal swabs. A wide panel of pathogen-specific antibodies were screened in serum, along with other pertinent measures such as routine clinical data and

physical assessments. Comparison of demographic and assessment data between time points suggested that health did decline in these participants, although it is not clear to me whether selection bias truly wasn't apparent (see below). This is critical given that the method of recruitment is very susceptible to this risk. The authors go on to report significant trajectories of routine biological measures, pathogen exposure, and disease incidence. They also report changes in serum PhenoAge over time, finding differences between time points when considering disease status, which I am assuming represents any co-morbid condition. These comparisons weren't exactly clear either, as detailed below. While the manuscript is very well written, and the research in exceptional in its magnitude, the findings mostly lack novelty, compounded by concerns regarding the selection bias. I also am not sure the title, which implies that "immunological variation" was studied here, really fits the data presented. My additional comments are below. Thank you for providing me the opportunity to review this work, I hope it is helpful.

We thank the reviewer for their comments on our manuscript and for acknowledging the 'exceptional research' presented here.

We understand the points raised regarding novelty and the appropriateness of the title. Both issues have now been addressed in the revised manuscript, as detailed in our point-by-point response below. Specifically, we now report the important finding that specific immune gene expression signatures, induced after whole blood stimulation with *Staphylococcus aureus* or *Candida albicans*, are predictive of diverse disease outcomes over a 10-year period. Notably, none of these immune genes differed between healthy and disease groups in the absence of immune stimulation, nor were significant differences observed in circulating immune cell phenotypes. This finding highlights the power of such an in depth, phenotyped longitudinal human cohort and underscores the potential of using functional immune responses as predictive biomarkers of disease development. <u>These results are now presented in a new Figure 6 and are a central focus of the revised Discussion.</u>

Comments

Line 211 – I am having trouble understanding the comparisons performed to discount potential selection bias for the cohort of 415. Figure 1B appears to be comparing the age of all participants at V1 vs. the participants at V2, showing age to be significantly higher, which is expected. What was the difference at V1 for those that ended up in V3 and those that did not? The comparison seems to be described this way for the SES factors, and does not appear to be different. Nonetheless, having a detailed summary table in the supplementary comparing demographic, lifestyle, SES and assessment measures at V1 for those that did and did not participate in V3 would be very helpful to truly be confident regarding the degree of selection bias (or lack thereof) for this follow-up study.

We thank the reviewer for raising this important point, which we have now addressed in the revised manuscript, specifically in Figure 1. To do so, we compared demographic variables at V1 between donors who were included in the V3 study and those who were not. While we did observe an age bias,

as originally reported, there were no significant differences in sex, BMI, smoking status, or socio-economic status—as assessed by an ELO score integrating housing status, income and education level. These variables were selected based on our previous work showing that they can significantly influence specific immune responses. This analysis supports the conclusion that there is no obvious demographic selection bias in the V3 recruitment.

Line 346 – The analysis of PhenoAge over time is very exciting, but the manner in which it is described in the text and reported in Figure 5 is unclear to me. It appears to indicate that higher PhenoAge at V1 is related to the incidence of disease at V3. Is that correct? How was that modelled? I am surprised that a change score model was not implemented wherein a participant's PhenoAge at V3 was regressed against their PhenoAge at V1 along with additional fixed effects representing other factors measured (e.g. SES, diseases, sex, etc.). Even serostatus for various pathogens could be included in such an analysis allowing for conclusions to be drawn regarding the role of infectious load in altering biological age trajectories.

We appreciate the reviewer sharing our enthusiasm for the Phenoage result, and we hope they find our new findings on immune response differences between healthy and disease groups at V1 even more compelling.

As requested, we have now clarified the specific analysis performed on Phenoage. First, we have simplified the analyses by comparing two groups, healthy and disease, to maximize statistical power and ease the interpretation.
The reviewer is correct that accelerated PhenoAge at V1 is associated with disease incidence at V3 (Figure 5c). This association was tested using a logistic model in which disease status at V3 was modelled as a function of PhenoAge at V1, adjusting for chronological age at V3. We have not reported results from models with additional fixed effects, as none of them were significantly associated with disease status. This has now been clarified in the revised Results section.

Reviewer #3 (Remarks to the Author):

In the manuscript "The Milieu Intérieur follow-up study – an integrative approach for studying immunological variation in an aging population," Jaquaniello et al. present results from a 10-year follow-up clinical study of a healthy cohort from the French population, aiming to identify biomarkers associated with the aging process. The study is well-designed and performed.

We thank the reviewer for their thoughtful comments and their positive appraisal of our study.

Below are some minor remarks:

• Although the authors present an approach to studying how the immune system changes with age, the manuscript would benefit from a more detailed discussion of their results and their implications for aging. For example, in the last sub-

section of the Results section, they mention that the "PhenoAge score was strongly correlated with chronological age," but the direction of changes in albumin, creatinine, glucose, and other biomarkers is not clear. It would also be interesting if the authors discuss how the observed changes can be related to health status and what their relation is to disease and aging itself.

We have now added the direction of changes for these biomarkers with age. Furthermore, we now report the changes in glucose levels with age in the new Figure 2. Finally, we have expanded the Discussion in the revised manuscript to address this point more thoroughly.

• There are statements in the Results section that are not linked to corresponding tables or figures. For example, in line 329, the authors state, "We found that the disease group was significantly older than the other groups (p=0.017)," but they do not cite the table in which these data are presented, making it unclear for the reader to see the exact difference between the groups. This issue occurs throughout the text, so please review and ensure proper citation of tables and figures.

This has been corrected in the revised manuscript.

• In lines 239 and 245, the authors present data from correlation analyses with false discovery rate correction of the p-values. This should be mentioned in the "Statistical analyses and data visualization" section.

Details have been added to the revised manuscript.

• The manuscript discusses physiological processes and biomarkers that can be influenced by various factors, including genetic background. Therefore, it is important to include information about the ethnicity of the cohort in the Materials and Methods section. Additionally, if the cohort consists of individuals from different ethnic backgrounds, this should be discussed accordingly.

We have now incorporated this information directly into the manuscript to address this point.

Reviewer #4 (Remarks to the Author):

I co-reviewed this manuscript with one of the reviewers who provided the listed reports. This is part of the NatureCommunications initiative to facilitate training in peer review and to provide appropriate recognition for Early Career Researchers who co-review manuscripts.

Thank you for your contribution.

REVIEWER COMMENTS

Reviewer #2 (Remarks to the Author):

Many thanks to the authors for considering my comments. I believe the new analyses and figures added do a nice job of confirming that selection bias wasn't a large issue.

Regarding the revisions to the PhenoAge analysis, I am still unclear what was done. In your rebuttal letter you state: "This association was tested using a logistic model in which disease status at V3 was modelled as a function of PhenoAge at V1, adjusting for chronological age at V3". First, it is common practice to report the odds ratio from a logistic regression model, and allows for comparability of estimates across studies. Second, although a logistic regression model was used, lines 277 and 288 report a t-test p-value. Please clarify. Lastly, the statement "We have not reported results from models with additional fixed effects, as none of them were significantly associated with disease status" is somewhat concerning given that one would expect variables such as sex, BMI and SES to be associated with the incidence of disease after ten years. If the authors can confirm that this is indeed the case, it would be important to mention as a limitation of this data, as it suggests the participants involved were likely derived from a low risk population and are not representative of the general population. This seems to be the case from the PhenoAge vs Chronological age scatterplot, since the entire cohort has a starting PhenoAge that is less than zero. I don't believe this is mentioned in the text, but please correct me if I am wrong.

- **We thank the reviewer for acknowledgeing our initial rebuttal and revisions and for their additional comments, which contribute to improve our manuscript.**
- **We now report odds-ratios throughout the main text, whenever relevant.**
- **Regarding the reporting of a *t*-test *p* value on 277, we apologize for this error, which has now been corrected; *t*-test has been replaced by Wald test. We have also carefully revised statistical tests in the entire manuscript and have corrected some omissions.**
- **We now clarify in the main text that the cohort derives from a low-risk population, providing a likely explanation (in addition to limited power) for the confirmed absence of significant associations between disease status and sex, BMI and SES.**

Reviewer #3 (Remarks to the Author):

I would like to thank the authors for their thorough and thoughtful revision of the manuscript. They have successfully addressed all the major and minor points raised in the previous round of reviews. The manuscript is now significantly strengthened, and the narrative is much clearer, more impactful, and better supported by the data presented. The manuscript, in its current form, contributes to our understanding of immune aging and the identification of biomarkers for future health outcomes.

We thank the reviewer for their very positive comments, which are greatly appreciated.

Reviewer #4 (Remarks to the Author):

I co-reviewed this manuscript with one of the reviewers who provided the listed reports. This is part of the Nature Communications initiative to facilitate training in peer review and to provide appropriate recognition for Early Career Researchers who co-review manuscripts.

- **We thank the reviewer for their contributions.**

Reviewer #5 (Remarks to the Author):

This is a well-written, revised manuscript detailing a number of analyses using an incredibly rich dataset from a well-characterized population. This cohort and it's data should provide unique opportunities to explore mechanisms of human aging. The revised manuscript includes several new analyses to bear further investigation.

The revised manuscript has several additions in response to reviewer concerns. Most of the reviewer concerns were addressed, although there are still some unanswered questions.
• Additional findings linking gene expression in response to microbial stimulation in vitro (a surrogate marker for immune functionality) with disease occurrence.
• A more complete V3 donor characterization. Results suggest that there is minimal selection bias.
• Virscan analysis highlighted several antibody responses to CMV proteins. Conclusion is that there is a general loss of Ab response to all antigens in participants who serorevert.

• Clarifications on the PhenoAge analysis. The response to reviewers indicates that the PhenoAge analysis did not include additional fixed effects because they were not associated with disease status. This is not explicitly stated in the section outlining PhenoAge results (lines 266-280).

• Results statements in the Discussion are still not linked to figures/tables.

- **Figures are now cited in the Discussion, when relevant.**

A new author was added to the revised manuscript. Most of the author change forms lack printed names and those signatures cannot be linked to the authors. Furthermore, several forms have the same signature(s) on them. These documents need to be resubmitted with printed names to ensure that all of the original authors agreed to the authorship change.

- **We have updated this document with printed names and all signatures, apologies for this previous oversight.**

  Was vaccination history between V1 and V3 collected? This may explain some of the immunologic changes. Also, why was mumps seroconversion so high? This was not discussed in the text and is at least as surprising as the similar B pertussis findings.

- **The thank the reviewer for this important comment. Vaccination history was collected in V1 though it was self-reported and previous analyses revealed that the collected data were not reliable (e.g., vaccination rates did not match national statistics; Scepanovic et al., Genome Med 2018). We therefore collected this information in V3 directly through vaccination cards. However the poor quality of the V1 data makes it challenging to compare vaccination rates between the two timepoints.**
- **Regarding the mumps seroconversion rate, there were 10 donors who were considered seronegative in V1, of whom 9 became seropositive. To support this result statistically, we have now revised these analyses, and discuss antigens with seroconversion rates significantly higher than 5% (Binomial test), which include mumps ($P_{adj} = 8.0 \times 10^{-10}$). We now comment these results in the main text and refer to a large serosurvey conducted in France, which reports evidence for mumps outbreaks (Béraud**

et al *Eurosurveillance* 2018). We thank the reviewer for their suggestion, which strengthens our manuscript.

Line 204-205 the authors suggest that their data support revaccination campaigns because there are changes in Ab titer over time. This would be a stronger statement if there was a decrease in seroprotection with age rather than simply 'dynamic changes in Ab titer with age'. Defined correlates of protection exist for several of the pathogens tested.

- **We agree with the reviewer that confirming loss of seroprotection would strengthen these statements. However the Luminex assays that were utilized to allow analysis of multiple serologies do not have validated correlates of seroprotection. Therefore we have modified this statement in the text to better reflect this point.**

The immune stimulation response being linked to disease development is one of the stronger and more interesting findings. The authors should discuss why some stimulations conditions seem 'better' (correlated with disease development) than others. Also, it is not the response to stimulus that is correlated with disease, it is the change in response to stimulus (V1 versus V3) that is correlated (See Figure 6a) – this needs to be more clearly stated in the Discussion.

- **We agree with the reviewer that these results are one of the most interesting findings from our study. We have added additional text to the discussion on why some stimulations may better capture this difference.**
- **We would like to clarify that it is actually the response to stimulus that is correlated with disease (i.e., we compared stimulated expression in V1 between diseased and healthy participants in V3), not the change between V1 and V3 responses. We have edited the text in the manuscript to make this point clearer.**

Table 3 – define 'at-risk'.

- **"At risk" referred to donors who recovered from a self-reported disease condition. To avoid confusion in the revised version, we now explicitly describe these participants as 'recovered' patients (Table3).**

REVIEWERS' COMMENTS

Reviewer #2 (Remarks to the Author):

Thank you for considering my comments. I am satified with the changes made and have no additional feedback.

Reviewer #5 (Remarks to the Author):

The authors have responded to all of the reviewer comments and have clarified the important points.

- **We thank the reviwers for their feedback**
